# Adaptive adipose tissue stromal plasticity in response to cold stress and antibody-based metabolic therapy

**DOI:** 10.1038/s41598-019-45354-1

**Published:** 2019-06-20

**Authors:** Joshua C. Chang, Steffen Durinck, Mark Z. Chen, Nadia Martinez-Martin, Jingli A. Zhang, Isabelle Lehoux, Hong Li, May Lin, Jiansheng Wu, Travis W. Bainbridge, James A. Ernst, Sree R. Ramani, Sairupa Paduchuri, Lance Kates, Margaret Solon, Matthew B. Buechler, Alessandra Castiglioni, Minh Thai, Beatrice Breart, Zora Modrusan, Andrew S. Peterson, Shannon J. Turley, Junichiro Sonoda

**Affiliations:** 1 Genentech, Inc., 1 DNA Way, South San Francisco, CA 90480 USA; 2 Present Address: Regeneron Pharmaceuticals, Inc., 777 Old Saw Mill River Rd., Tarrytown, NY 10591 USA

**Keywords:** Antibody therapy, Metabolic syndrome, Fat metabolism, Obesity

## Abstract

In response to environmental and nutrient stress, adipose tissues must establish a new homeostatic state. Here we show that cold exposure of obese mice triggers an adaptive tissue remodeling in visceral adipose tissue (VAT) that involves extracellular matrix deposition, angiogenesis, sympathetic innervation, and adipose tissue browning. Obese VAT is predominated by pro-inflammatory M1 macrophages; cold exposure induces an M1-to-M2 shift in macrophage composition and dramatic changes in macrophage gene expression in both M1 and M2 macrophages. Antibody-mediated CSF1R blocking prevented the cold-induced recruitment of adipose tissue M2 macrophages, suggesting the role of CSF1R signaling in the process. These cold-induced effects in obese VAT are phenocopied by an administration of the FGF21-mimetic antibody, consistent with its action to stimulate sympathetic nerves. Collectively, these studies illuminate adaptive visceral adipose tissue plasticity in obese mice in response to cold stress and antibody-based metabolic therapy.

## Introduction

Visceral adiposity, rather than subcutaneous adiposity, shows a strong correlation with insulin resistance and plays a central role in the pathogenesis of obesity-related diseases^1–3^. In healthy visceral adipose tissue (VAT), anti-inflammatory type 2 immune cells such as adipose tissue M2 macrophages (ATM2), eosinophils and group 2 innate lymphoid cells are dispersed throughout the tissues^4,5^. Genetic deletion of type 2 cytokines or depletion of group 2 innate lymphoid cells leads to adipose tissue inflammation and enhanced susceptibility to insulin resistance, highlighting the importance of type-2 immunity in adipose tissue homeostasis^5–7^. Over-nutrition in obesity leads to a saturation of lipid storage in VAT adipocytes, causing adipocyte death and recruitment of inflammatory adipose tissue M1 macrophages (ATM1) to a “crown-like structure” (CLS)^4,8–10^. ATM1, together with other inflammatory immune cells in obese VAT, are thought to contribute to insulin resistance by producing inflammatory cytokines such as TNFα, IL1β, and RELMα^11–14^.

Adipose tissues are under the neural control of the sympathetic nervous system (SNS), mediated by tyrosine hydroxylase (TH)-positive catecholaminergic neurons that innervate from the paravertebral sympathetic ganglia into adipose tissues^15–17^. Physiological stress such as cold exposure stimulates sympathetic nerves to release catecholamine, which then activates adrenergic receptors expressed in adipocytes and stromal cells to trigger lipolysis, adaptive thermogenesis, and white adipose browning^15,17,18^. Cold exposure also stimulates sympathetic nerve branching, suggesting the existence of a positive-feedback regulation^19,20^, although the mechanism underlying this phenomenon is not understood. The role of ATM2 and other type 2 immune cells in the cold-induced browning of inguinal subcutaneous adipose tissue (SCAT) in lean healthy mice has been documented^6,21–23^. Adipose browning can also be observed in VAT after non-physiological exposure to catecholamine in humans with pheochromocytoma or in mice exposed to adrenergic β3-selective agonist, suggesting the presence of pre-existing adipogenic progenitor (AP) cells that can differentiate into UCP1^+^ beige adipocytes^24–29^. However, cold-induced adipose browning is generally absent in healthy VAT in lean mice^23,26^, which could be attributed to a scarcity of sympathetic nerve fibers and lesser cold-induced SNS drive in this tissue^19,30^. These studies overall implicated a therapeutic SNS stimulation in the treatment of obesity-associated insulin resistance; however, the consequence of the SNS stimulation in VAT microenvironment in obese animals is poorly understood, motivating us to interrogate the effect of cold-exposure and a drug-induced SNS stimulation in obese VAT phenotype. Here, we describe a dynamic visceral adipose tissue stromal remodeling in response to the SNS stimulation, that involves adipose tissue macrophages.

## Results

### Cold exposure induces VAT remolding in obese mice

**Figure 1:**
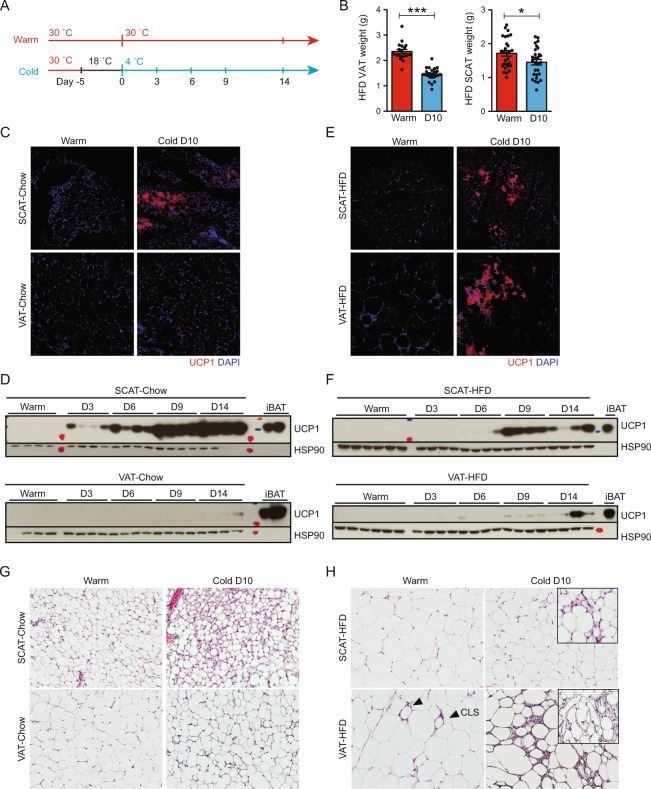
Cold exposure induces weight loss and browning in obese mice. C57BL/6 mice on either normal chow (10% kcal fat) or HFD (60% kcal fat) were maintained at thermoneutrality (30**°**C, Warm) or exposed to 4°C (Cold) after adaptation at 18**°**C. (**A**) The design of the experiment. (**B**) VAT and SCAT weights after 10-days 4°C cold exposure (N ≥ 18). *P < 0.05, ***P < 0.001 by Student’s *t*-test. Data are shown as mean ± SEM. (**C**) Representative IF for UCP1 (red) in lean SCAT and VAT. (**D**) Western blot analysis for UCP1 expression in lean mice maintained at thermoneutrality or exposed to 4**°**C for an indicated time as in Fig. 1A. HSP90 serves as loading control. (**E**) Representative IF for UCP1 (red) in HFD-fed SCAT and VAT. DAPI (blue) stains cell nuclei. (**F**) Western blot analysis for UCP1 expression in obese mice maintained at thermoneutrality or exposed to 4**°**C for an indicated time as in Fig. 1A. HSP90 serves as loading control. (**G**) H&E staining in lean SCAT and VAT. (**H**) H&E staining in HFD-fed SCAT and VAT. CLS in Warm VAT are indicated by triangles. Inlets highlight separate areas within the same section exhibiting multilocular beige adipocytes.

To examine the VAT response to cold exposure, C57BL/6 mice on either standard chow (Chow: 10% kcal fat) or a high-fat diet (HFD: 60% kcal fat) maintained at a thermoneutral temperature of 30 °C were exposed to 4 °C (Cold) after a 5-day acclimation period at 18 °C. Control (Warm) mice were kept at 30 °C throughout the study to minimize cold stress (Fig. 1A). Upon cold exposure, chow-fed lean mice maintained body weight while food consumption increased by nearly 100% (Figure S1A-B). Cold-exposed HFD-fed diet-induced obese (DIO) mice, in contrast, showed a significant weight loss and an improvement in various metabolic markers despite a ~30% increase in food intake (Figure S1C-D). Notably, the decrease in epididymal VAT weight (38%) was more pronounced as compared to inguinal SCAT (15%) in HFD-fed obese mice after 10-days 4 °C cold exposure (Fig. 1B). As expected, adipose tissue browning characterized by the emergence of UCP1^+^ multilocular adipocytes and increased UCP1 protein expression by western blot was observed in SCAT, but not in VAT in the lean animals (Fig. 1C,D). Unexpectedly, we could detect rare patches of UCP1^+^ multilocular adipocytes throughout VAT, in nearly half of the obese animals exposed to cold, although it was more pronounced in SCAT (Fig. 1E). We also observed a slight but notable expression of UCP1 protein in obese VAT in addition to SCAT by western blot (Fig. 1F). *Ucp1* mRNA expression was highly induced by cold exposure in obese BAT and SCAT **(**Figure S1E). We also observed a trend in *Ucp1* mRNA induction in obese VAT, but it did not reach statistical significance **(**Figure S1E). Hematoxylin and eosin (H&E) stained tissue sections confirmed the cold-induced emergence of multilocular beige adipocytes in lean SCAT, and to a lesser extent in obese SCAT (Fig. 1G,H). This was absent in cold-exposed lean VAT. In warm obese VAT, the presence of CLS was evident. After cold exposure, multilocular beige adipocytes emerged as patches in obese VAT (Fig. 1H). More significantly, cold-exposed obese VAT exhibited an unexpected presence of thick layers of stromal cells clustered throughout the tissue (Figs 1H and S1F).

**Figure 2:**
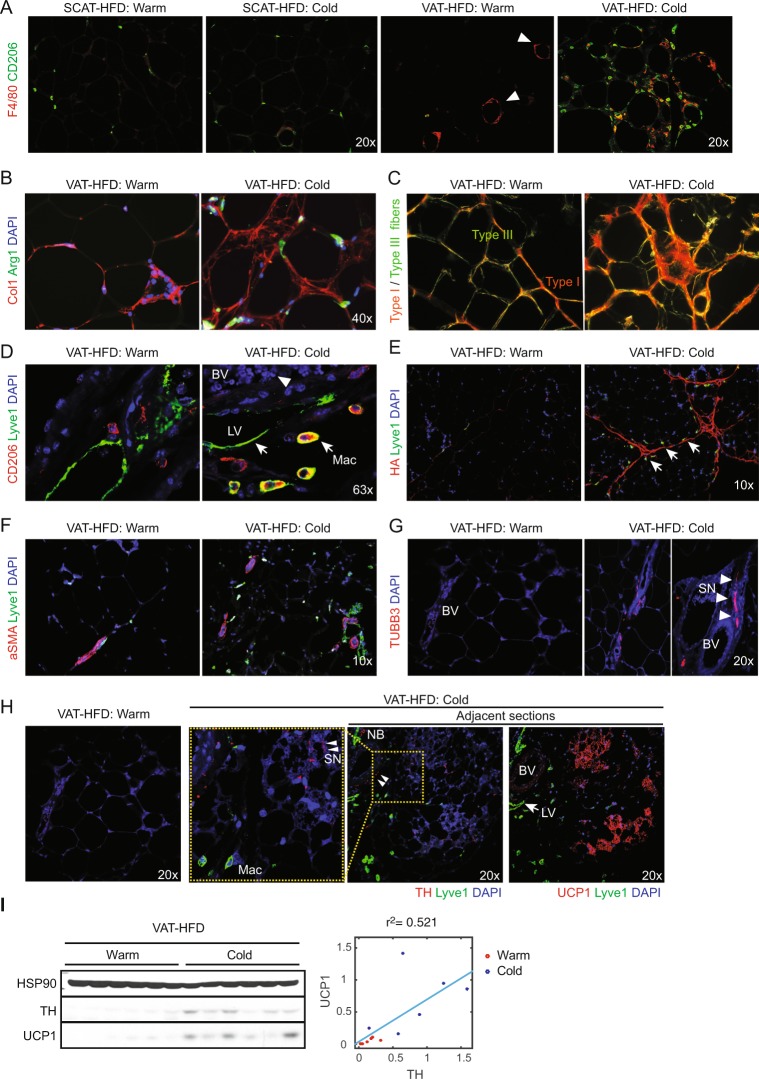
Cold exposure induces VAT remolding in obese mice. HFD-fed mice were maintained at 30**°**C (Warm) or exposed to 4**°**C for 10 days (Cold) as indicated in Fig. 1A. Representative IF in HFD-fed SCAT (**A**) and VAT (**A**–**H**) were shown. (**A**) CD206 (green) and F4/80 (red) IF. CLS are indicated by triangles. (**B**) Collagen I (red) and Arg1 (green) IF. (**C**) Picro Sirius Red staining imaged with polarized light microscopy, visualizing collagen fibers type I (thick fibers, yellow-orange) and type III (Thin fibers, green). (**D**) CD206 (red) and Lyve1 (green) IF. Red blood cells (arrow), Lyve1^+^ ATM2 (Mac), Lyve1^+^ lymphatic vessels (LV) are indicated. (**E**) Hyaluronan fibers (HABP-stain, red) and Lyve1 (green) IF. Arrows indicate ATM2 (**F**) aSMA (red) and Lyve (green) IF. (**G**) TUBB3 (red) IF. Sympathetic nerves (SN) are indicated by triangles, surrounding blood vessel (BV). Higher magnification image is shown at side. (**H**) Lyve1 (green) and TH (left) or UCP1 (right) (red) IF in adjacent sections (right two). Higher magnification images of the inlets (yellow dashed boxes) are also shown at left side. Note TH^+^ nerves (triangles) were closely associated with beige adipocytes (red) and blood vessels (BV). NB, nerve bundles. DAPI (blue) stains cell nuclei. For each IF analysis, at least 10 independent mice per group were examined, and representative images are shown. (**I**) Western blot analysis for TH, UCP1, and HSP90 (loading control). At right, quantified TH and UCP1 expression are plotted to show correlation.

CLS with F4/80^+^ ATM were abundant in obese VAT, but not evident in SCAT under warm condition (Fig. 2A). In obese VAT, but not in SCAT, cold-exposure induced dense clusters of F4/80^+^ ATM that were distinct from thin CLS (Fig. 2A). Many of these ATM were positive for ATM2 marker arginase 1 (Arg1) and closely associated with collagen I (Col1) surrounding adipocytes in cold-exposed obese VAT (Figs 2B and S2A). Picro Sirius Red staining followed by polarized light imaging revealed that ECM encircling obese VAT adipocytes is mostly composed of thin fibrils of type III collagen, whereas cold exposure induced an accumulation of closely packed thick fibrils of type I collagen (Fig. 2C). The lymphatic vessel endothelial hyaluronan receptor 1 (Lyve1) was detected on the surface of the lymphatic vessels but also expressed by most of CD206^+^ ATM2 (Figs 2D and S2B). Intriguingly, the majority of these Lyve1^+^ ATM2 lined up along the cold-induced hyaluronan fibers as if they were migrating in rows (Figs 2E and S2C). In addition, α smooth muscle actin (αSMA)-positive blood vessels were more prevalent after cold exposure in obese VAT (Figs 2F and S2D). In warm obese VAT, nerve fibers that are positive for dopaminergic marker tyrosine hydroxylase (TH) are rare, but after cold-exposure, nerve fibers that are positive for dopaminergic marker TH or pan-neuronal marker TUBB3 become more numerous (Fig. 2G,H). They are almost always bundled along with blood vessels as thin fibers (Fig. 2G,H, triangles) or thick bundles of fibers (nerve bundles, NB), and can also be seen frequently scattering around newly formed UCP1^+^ adipocytes and Lyve1^+^ ATM2 (Fig. 2H), consistent with previous reports that demonstrated cold-induction of TH^+^ sympathetic nerve branching in lean VAT^19,20^. An examination of UCP1 and TH protein expressions in HFD-fed VAT tissues revealed a cold-induction of TH protein expression that positively correlated with the UCP1 protein expression (Fig. 2I). These observations together reveal a previously unappreciated obese VAT remodeling in response to cold-induced sympatho-stimulation.

### CSF1R-dependent recruitment of ATM2 into cold stimulated obese VAT

**Figure 3:**
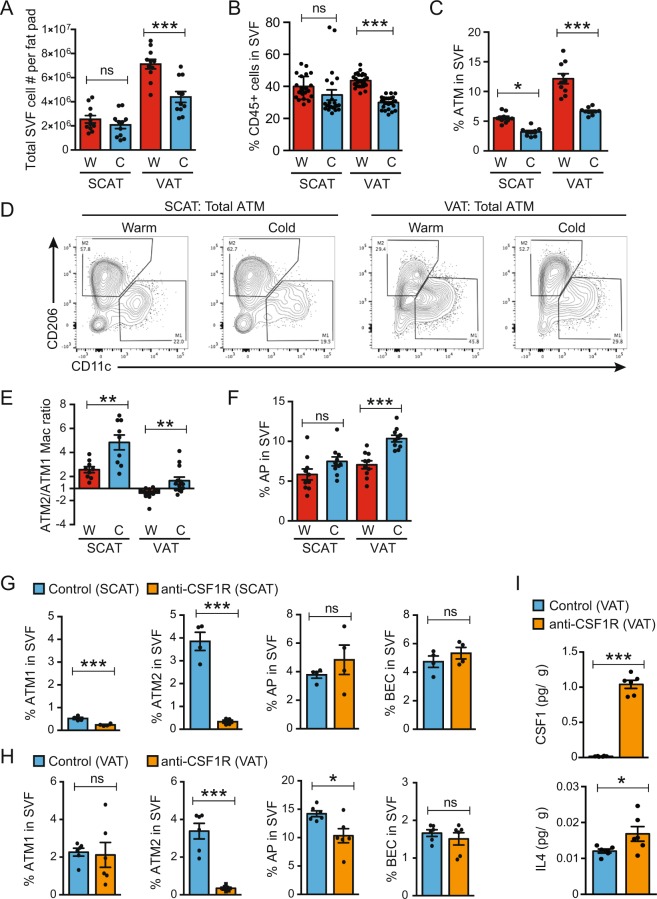
Cold exposure recruits newly differentiated ATM2. HFD-fed mice were maintained at 30**°**C (W: Warm) or exposed to 4**°**C for 10 days (C: Cold) as indicated in Fig. 1A. (**A**) Total SVF cell count per fat pad. (**B**) Frequencies of CD45^+^ cells in live SVF. (**C**) Frequencies of CD45^+^/F4/80^+^/CD11b^+^/Gr1^−^/Fcer1^−^/siglec-f^−^ total ATM in total SVF. (**D**) Representative plots for M2/M1 ATM populations. (**E**) Ratio of the frequencies of ATM2 (CD11c^−^/CD206^+^) over ATM1 (CD11c^+^/CD206^−^). (**F**) Frequencies of CD45^−^/CD31^−^/PDGFRα^+^/Sca1^+^/CD29^+^ AP in SVF. (**G–I**) The effects of anti-CSF1R antibody treatment in HFD-fed mice during cold exposure, using the same gating strategy as above. The frequencies of ATM1, ATM2, AP, and BEC in SCAT (**G**) and VAT (**H**) in SVF. (**I**) Cytokine concentrations in VAT lysate. *P < 0.05, **P < 0.01, ***P < 0.001 by Student’s *t*-test. Data are shown as mean ± SEM.

To validate the histological observations described above in a quantitative manner, flow cytometry analysis was utilized to examine the stromal vascular fraction (SVF) isolated from SCAT and VAT in HFD-fed mice with or without cold exposure (Figure S3A). Warm mice showed nearly three times as many SVF cells in VAT than in SCAT (Fig. 3A). Cold exposure reduced SVF cell numbers per fat pad and the frequency of CD45^+^ hematopoietic cells in VAT but not in SCAT (Figs 3A,B and S3B). The frequency of total F4/80^+^/CD11b^+^/Gr1^−^/FceR1^−^/Siglec-f^−^ ATM in warm obese VAT was ~2-fold higher than in SCAT, and cold exposure decreased it by half in both tissues (Figs 3C and S3C). Cold exposure also reduced CD11c^+^/CD206^-^ ATM1 population and conversely expanded CD11c^−^/CD206^+^ ATM2 population in both tissues (Figs 3D and S3D**)**. As a result, the ATM2/ATM1 ratio in obese VAT was reversed from 0.8 (i.e., ATM1 dominant) to 1.7 (i.e., ATM2 dominant), suggesting a reversal of meta-inflammation (Fig. 3D,E). The increase in ATM2 could be due to an increase in monocyte recruitment or macrophage differentiation within the tissue. In SCAT, ATM2 predominated in both warm and cold-exposed conditions (Figs 3D and S3D-E). Consistent with the histological studies, cold exposure increased CD45^−^/PDPN^−^/CD31^+^ blood endothelial cells (BEC), indicative of angiogenesis in obese VAT, but not in obese SCAT (Figure S3E). The frequency of CD45^+^/F4/80^−^/CD11c^+^ dendritic cells did not change in either tissue (Figure S3F). UCP1^+^ beige adipocytes in cold-exposed obese VAT emerged as rare clusters, suggesting de novo differentiation from proliferative AP cells. The CD45^-^/CD31^-^/PDGFRα^+^/Sca1^+^/CD29^+^ cells have been identified as bipotential AP in VAT that can differentiate into either white or beige adipocytes^25,31^. We found a significant increase in the frequency of AP in cold-exposed obese VAT, but somewhat surprisingly, not in SCAT (Figs 3F and S3G). Expression of an inflammation-inducible protein, Podoplanin (PDPN)^32^ in AP was higher in VAT than in SCAT and decreased upon cold exposure, which is consistent with a notion that cold exposure attenuates meta-inflammation in obese VAT (Figure S3H).

Colony-stimulating factor 1 (CSF1) and its receptor, CSF1R, regulate the migration, differentiation, and survival of macrophages and their precursors^33^. In order to test the requirement for CSF1R signaling in the observed ATM2 recruitment, we utilized a blocking monoclonal antibody against for CSF1R (anti-CSF1R)^34,35^ (Figure S4A). We found that a systemic administration of anti-CSF1R diminished the frequency of ATM2 in obese SCAT and VAT (Figs 3G,H and S4B,C), especially the Lyve1^+^ ATM2 (Figure S4C-D). In contrast, ATM1 was reduced in SCAT but not in VAT. In response to anti-CSF1R administration, the cytokines CSF1 and interleukin-4 (IL4) in VAT lysates were elevated, suggesting a feedback regulation of these cytokines (Fig. 3I). We also observed a modest decrease in the frequency of AP in obese VAT, but not in obese SCAT after the anti-CSF1R treatment (Figs 3G,H and S4E-F), which is likely an indirect effect since CSF1R is expressed by ATM but not by AP (Figure S4G). Anti-CSF1R treatment did not appreciably affect cold-induction of UCP1 protein expression, although it eliminated signal for CD206 as anticipated (Figure S4H). Thus, the SCAT browning does not depend on the ATM2 in obese mice. In VAT, the results were less clear, probably in part due to the inconsistency in the cold-induced UCP-induction in this tissue (data not shown). Anti-CSF1R also increased the circulating level of pro-inflammatory cytokines such as IL1α and IL12p70 (Figure S4I**)**. These results together indicated that the intact CSF1-CSF1R signaling is vital for regulating macrophage population in the adipose tissues during cold exposure, although it may not be required for adipose tissue browning.

### The effect of cold exposure in ATM and AP

**Figure 4:**
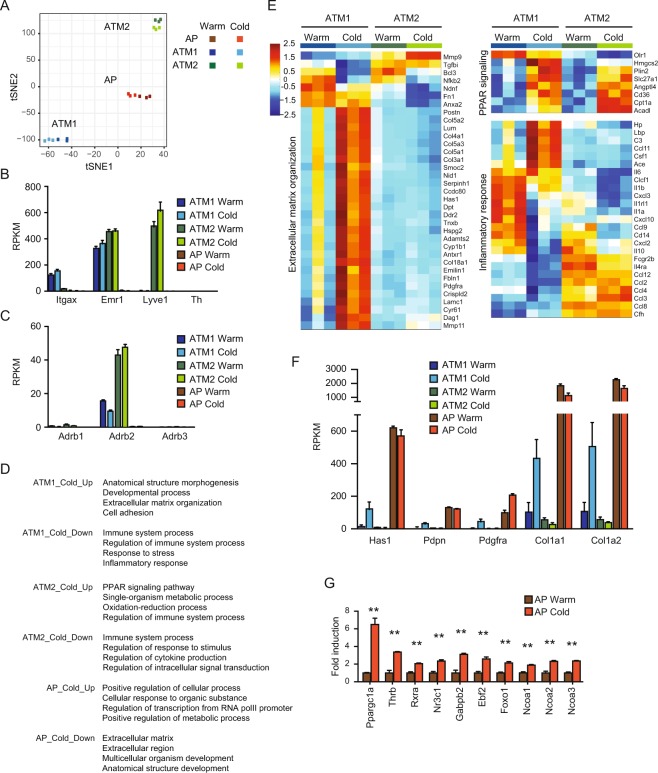
ATM and AP gene expression after cold exposure. Gene expression analysis in obese VAT ATM and AP: HFD-fed mice were maintained at 30**°**C (Warm) or acclimated at 18**°**C for 7 days before exposure to 4**°**C for 8 days (Cold). ATM1, ATM2 and AP were FACS sorted from VAT for RNA-Seq analysis. (**A**) tSNE analysis. (**B**) The expression of *Itgax, Emr1, Lyve1*, and *Th*. (**C**) The expression of adrenergic receptor genes. (**D**) Gene Ontology (GO) enrichment analysis of differentially expressed genes between warm and cold conditions in ATM1, ATM2, and AP. (**E**) Heatmap showing ATM expression of various genes in indicated GO pathways (ECM organization: GO 0030198; PPAR signaling pathway: KEGG 03320; Inflammatory response: GO 0006954). For expression heatmaps, data (log_2_(RPKM)) are standardized along each gene (row), and color scale corresponds to the relative expression level. (**F**) Expression of ECM-related genes that were regulated by cold-exposed in ATM1. (**G**) Induction of transcription activator genes implicated in beige/brown adipogenesis in AP. **P < 0.01 by Student’s *t*-test. Data are shown as mean ± SEM (N = 3 samples, 3 mice pooled as one sample).

The conversion of the ATM population from M1-dominant to M2-dominant in cold-exposed obese VAT suggested us the role of ATMs in regulating tissue inflammation and remodeling in obese mice. The observed obese VAT browning also suggested the involvement of AP in the tissue remodeling. Thus, we conducted RNA-Seq analysis in FACS-sorted ATM1, ATM2 and AP isolated from obese VAT in warm and cold conditions. Cells were isolated from VAT in HFD-fed mice maintained at 30 **°C** (Warm) or acclimated at 18 **°C** for 7 days before exposure to 4 **°**C for 8 days (Cold) using the same gating strategy as described above. Gene expression profile in each of the three cell types was separated into distinct clusters with clear separation between the warm and cold groups in the t-distributed stochastic neighbor embedding (tSNE) analysis (Fig. 4A). ATM1 and ATM2 both express *Emr1* (F4/80); whereas ATM1 and ATM2 specifically express *Itgax* (CD11c) and *Lyve1*, respectively, consistent to the IF staining and FACS analysis (Fig. 4B). A comparison between ATM1 and ATM2 under thermoneutrality defined the top 30 “ATM1 genes” and the top 30 “ATM2 genes”. Cold exposure did not significantly alter the expression of these genes (Figure S5A). Interestingly, *Adrb2* gene encoding β2 adrenergic receptor was also detected in ATM1 and ATM2, indicating a potential regulation these macrophages by catecholamines (Fig. 4C).

Gene ontology (GO) analysis revealed cold-induced upregulation of ECM-related genes in ATM1, upregulation of PPARγ/δ target genes in ATM2, and downregulation of immune system process genes in both ATM1 and ATM2 (Fig. 4D,E). In particular, ATM1 and ATM2 showed differential inflammatory gene expression in response to cold exposure, such as down-regulation of *Il1a*, *Il1b*, *Retnla* (encoding RELMα), *Mgl2* (encoding CD301b) and upregulation of *Il33*, *Ccl11*, *Csf1* and *Spp1* (encoding osteopontin), suggesting a Type-1-to-Type-2 shift in tissue inflammation (Fig. 4E). In addition, ATM1 also upregulated expression of *Hyaluronan synthase 1* (*Has1*), *Collagen 1a* (*Col1a*) and other ECM genes, thus may contribute to the recruitment of Lyve1^+^ ATM2 via binding of Lyve1 to hyaluronan-containing ECM, although AP expressed genes encoding *Hyaluronan synthase 1* (*Has1*) and *Collagen 1a* (*Col1a*) at higher levels (Fig. 4F).

GO analysis also revealed cold-induced alternations in transcription factors and ECM-related genes in AP (Figs 4D and S5B). In particular, the expression of genes encoding a number of transcription factors and cofactors known to be important for beige/brown adipocyte differentiation and mitochondrial function^36^ were induced by cold exposure, consistent with the role as the origin of UCP1^+^ beige adipocytes (Fig. 4G).

### Possible roles of BMP/GDF ligands in cold exposed obese VAT

**Figure 5:**
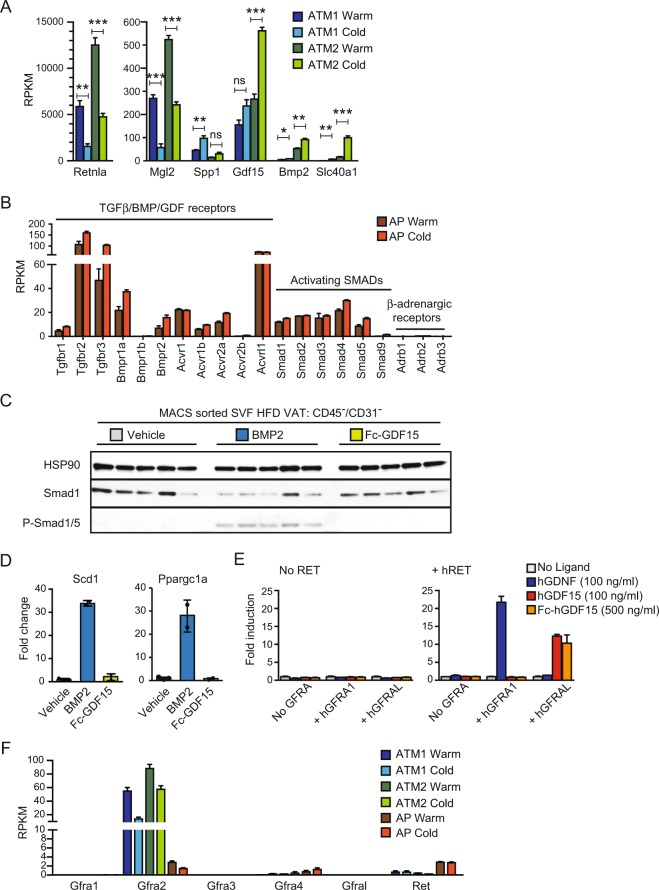
The potential role of GDF/BMP ligands in AP differentiation. (**A**) ATM Genes regulated by cold exposure. *P < 0.05, **P < 0.01, ***P < 0.001 by Student’s *t*-test. (**B**) The expression of BMP receptor and Smad genes in AP. (**C**) Western blot analysis in sorted CD45^−^/CD31^−^ cells from HFD-fed VAT treated with the indicated ligand at 500 ng/ml for 30 min. HSP90 expression serves as loading control (N = 5). (**D**) qPCR analysis of *Scd1* and *Ppargc1a* expression in FACS sorted AP from HFD-fed VAT after adipocyte differentiation. Cells were differentiated for 14 days in the presence or absence of the indicated ligand at 250 ng/ml. (**E**) GAL-ELK1 luciferase assay in HEK293T cells to monitor RET stimulation. Cells were transfected to express the appropriate luciferase reporter construct and an appropriate GFRA coreceptor as indicated with (right) or without (left) RET. Transfected cells were stimulated with the indicated ligand. Results shown as mean fold induction ± SEM (N = 3). (**F**) Expression of *Gfra* and *Ret* receptor genes in warm and cold conditions in FACS sorted ATM1, ATM2, and AP. Data are shown as mean ± SEM (N = 3).

Previously, ATM2-derived catecholamine was proposed to drive beige adipogenesis^22^. However, we failed to detect expression of *Th* gene, encoding the rate-limiting enzyme, tyrosine hydroxylase, responsible for catecholamine production (Fig. 4B). To identify ATM2-derived factors that could instruct AP differentiation into beige adipocytes, we looked for abundantly expressed genes (RPKM (Reads per kb per million mapped reads) > 40 in cold ATM2) encoding a secreted protein whose expression was elevated selectively in ATM2. This analysis identified only two candidate genes, *Gdf15* and *Bmp2* (Fig. 5A). ATM2 also induced expression of *Slc40a1*, encoding the only known mammalian iron-exporting protein Ferroportin (Fig. 5A), which may contribute to an increase in local iron concentration and mitochondria biogenesis. GDF15 and BMP2 were attractive candidates as ATM2-derived factors that augment VAT browning since they belong to the TGFβ/BMP/GDF ligand family whose general function is to induce cell differentiation by activating the Smad signaling pathways^37,38^. Indeed, AP exhibited significant expression of genes encoding candidate receptors that might be targeted by BMP2 or GDF15 (Fig. 5B). In contrast, no expression of β-adrenergic receptors was detected in AP (Fig. 5C). When isolated CD45^-^/CD31^-^ stromal fibroblasts containing AP (>70%) were treated with either BMP2 or GDF15, only BMP2 induced Smad1/5 phosphorylation (Fig. 5C), and mRNA expression of *Ppargc1a* and lipogenic *Scd1* genes (Fig. 5D). Although previously reported to activate canonical TGFβ signaling^39^, GDF15, a long-known orphan BMP-like ligand with neurotrophic activity^40^, was recently reported to act as a GDNF-family ligand that stimulates the receptor tyrosine kinase RET via binding to the coreceptor GFRAL (GDNF Family Receptor Alpha-Like) expressed in the brainstem neurons^41–44^. We have independently identified GFRAL as a binding partner for GDF15 (not shown) and found that only GDF15, but not GDNF, can activate GAL-ELK1 reporter in HEK293T cells expressing GFRAL and RET (Fig. 5E). However, neither *Ret* or *Gfral* mRNA expression was detected in ATM or AP (Fig. 5F).

### Administration of FGF21-mimetic antibody phenocopies cold exposure in obese VAT

**Figure 6:**
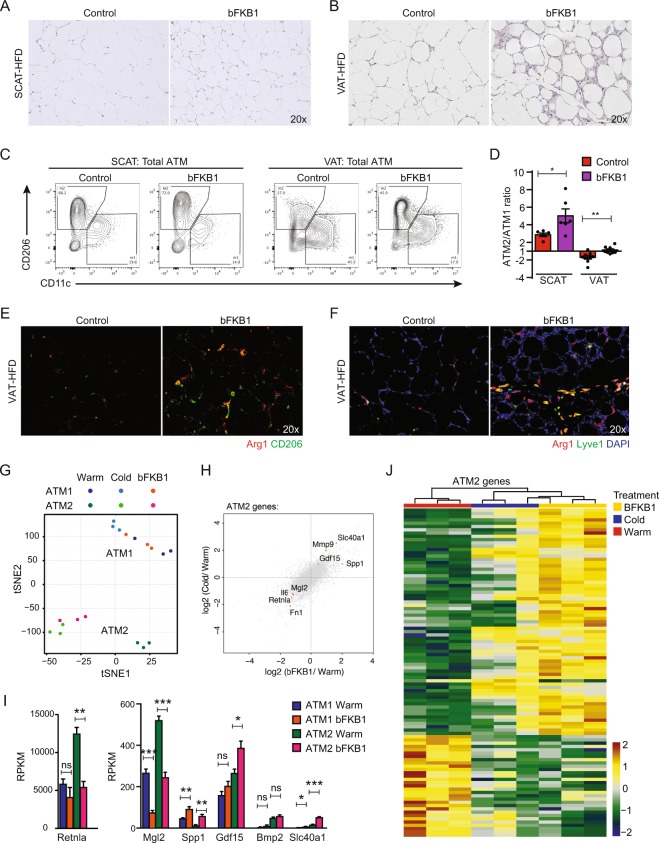
Administration of FGF21-mimetic antibody phenocopies cold exposure in obese VAT. HFD-fed male mice received bFKB1 (10 mg/kg i.p.) or vehicle once on day 0 at thermoneutral condition. Tissues were harvested on day 14 after bFKB1 injection for histology and FACS analysis (**A**–**F**), and day 8 for gene expression analysis (**G**–**J**). (**A**) H&E staining in HFD-fed SCAT. (**B**) H&E staining in HFD-fed VAT. (**C**) Representative plots for M2/M1 macrophage populations. (**D**) Ratio of the frequencies of ATM2 (CD11c^−^/CD206^+^) over ATM1 (CD11c^+^/CD206^−^). (**E**) CD206 (green) and Arg1 (red) IF. (**F**) Lyve1 (green) and Arg1 (red) IF. DAPI (blue) stains cell nuclei. (**G**) tSNE analysis of RNA-Seq results from ATM1 and ATM2 from obese VAT. (**H**) 4-way differential analysis revealed bFKB1-induced gene expression profile in ATM2 mimics cold exposure. Grey dots indicate all the expressed genes. Red dots indicate genes of interest. (**I**) Expression of genes regulated by bFKB1 in ATM1 or ATM2. Data are shown as mean ± SEM (N = 3 samples, 5 mice pooled as one sample). (**J**) Heatmap showing the expression of bFKB1-regulated genes in ATM2. The gene set was selected based on a 2-Way comparison between bFKB1-treated samples and control (warm) samples. Color scale corresponds to the relative expression level (log_2_(RPKM) standardized along each gene). The gene names are provided in Figure S9. *P < 0.05, **P < 0.01, ***P < 0.001 by Student’s *t*-test.

Recombinant fibroblast growth factor 21 (FGF21) protein stimulates brown fat thermogenesis, improves metabolic health, induces SCAT browning and weight loss when administered to obese mice^45–47^. The action of recombinant FGF21 can be mimicked by the anti-Fibroblast Growth Factor Receptor 1 (FGFR1)/βKlotho bispecific antibody bFKB1 that activates the FGFR1/βKlotho receptor complex, the primary target of FGF21^48,49^. Because of the similarities in the metabolic effects of cold exposure and those of the administration of FGF21 or bFKB1, we wondered whether these FGF21-class molecules also induce the VAT stromal remodeling similar to cold exposure. To test this idea, we administered bFKB1 into DIO mice housed at thermoneutral temperature. Indeed, bFKB1 induced obese adipose tissue remodeling phenotype similar to that induced by cold exposure (Fig. 6A,B). By flow cytometry, bFKB1 also increased the prevalence of ATM2 population (Fig. 6C,D). The recruitment of ATM2 was also confirmed by IF with Arg1, CD206, and Lyve1 markers in the bFKB1 treated obese VAT (Fig. 6E,F). RNA-Seq followed by tSNE analysis and differential analysis revealed a striking similarity between cold-induced and bFKB1-induced gene expression changes in ATM2 (Fig. 6G,H). We identified genes that are commonly upregulated by both cold exposure and bFKB1 in obese VAT ATM2, including *Gdf15* and *Slc40a1* (Fig. 6H,I). In order to further examine the relationship between bFKB1-treatment and cold exposure in regulating ATM2 gene expression, we generated a heatmap that includes all three conditions (Fig. 6J). The gene set was selected based on a 2-Way comparison between bFKB1-treated samples and control (warm) samples. Hierarchical clustering analysis revealed the similarity between bFKB1-treated samples and cold-exposed samples, as we expected from the tSNE-analysis (Fig. 6G) and the 4-Way comparisons (Fig. 6H). These results demonstrate the action of bFKB1 to mimic cold exposure in inducing VAT remodeling and regulating macrophage gene expression.

## Discussion

Our work illuminates the ability of the cold-induced and pharmacologic sympatho-stimulation in obese VAT to recruit ATM2 and alleviate chronic meta-inflammation while inducing tissue remodeling and rare adipose tissue browning. Although we believe that the cold-induced sympathetic stimulation in the obese VAT has significant metabolic consequences, the modest induction of UCP1-positive beige adipocytes in VAT is unlikely sufficient to contribute much to overall thermal defense or whole-body energy metabolism. Our current hypothesis is that cold-induced reversal of meta-inflammation likely contributes to the cold-induced improvement in metabolic health. The cold-induced immune alternations and tissue remodeling that are evident in obese VAT are unnoticeable in obese SCAT, which is characterized by a paucity of inflammatory macrophages, implicating the role of inflammatory cells in this process. In addition to decreasing the expression of inflammatory cytokines implicated in inflammation-induced insulin resistance and hyperglycemia^12,13^, cold exposure also increases the expression of various type 2 cytokines and ECM-components and enzymes, such as collagens, lysyl-oxidases, and the hyaluronan-producing enzyme hyaluronan synthase 1 (*Has1*) in ATM1. The newly recruited ATM2 in the cold-exposed obese VAT are marked by the hyaluronan receptor Lyve1 and closely associated with newly produced hyaluronan fibers, thus suggesting possible mechanisms by which ATM1 and hyaluronan fibers contribute to the ATM2 recruitment.

Our work revealed that the cold-induced ATM2 recruitment in obese VAT is dependent on the CSF1R-mediated signaling implicated in macrophage differentiation. The number of APs was increased in VAT but not in SCAT after cold exposure. Concomitantly, anti-CSF1R treatment reduced the number of AP in VAT, but not in SCAT, suggesting that the cold-induction in AP number in VAT is dependent on CSF1R signaling. One possibility is that the AP number is suppressed in obese VAT by the tissue inflammation. Obese VAT is ATM1-dominant, which can be converted to ATM2 dominant by cold exposure. On the other hand, ATM2 is dominant in obese SCAT even in warm condition. Previously, clodronate liposome- or CD206-DTR-induced macrophage depletion were used to test the role of adipose tissue macrophages in regulating cold-induced SCAT browning in lean mice with conflicting results^22,23,50,51^. In our study with DIO mice, SCAT browning was not visibly affected by anti-CSF1R treatment. Thus, the role of macrophages in adipose browning may be complex and context dependent. Interestingly, TH expression was not found in ATM1, ATM2, nor AP, but the expressions of adrenergic receptors were clearly detected in ATM1 and 2, suggesting a direct role of the sympathetic nerves to regulate immune cell function during VAT remodeling. The observation of cold-induced VAT macrophage polarization is evocative of a recently described sympathetic regulation of intestinal macrophages during enteric infection^52^, thus may represent a general mode of neuro-immune communication.

Our study also revealed the action of bFKB1, an FGF21-mimetic anti-FGFR1/βKlotho antibody, to induce VAT remodeling in obese mice that closely resemble the cold-induced visceral adipose remodeling. Previously, administration of recombinant FGF21 protein in mice was shown to stimulate sympathetic nerves innervating brown adipose tissues^53^. Consistent with the action on the nervous system, FGF21 and bFKB1 were both shown to act via βKlotho co-receptor expressed in the nervous system, rather than in adipocytes, to induce weight loss and improved glucose tolerance^48,53,54^. Our result supports the notion that FGF21 and bFKB1 both act primarily by stimulating sympathetic nerves to ameliorate tissue inflammation, induce browning, and stimulate brown/beige fat thermogenesis. FGF21-receptor agonists including PEGylated FGF21, Fc-FGF21 fusion, and FGF21-mimetic antibodies are currently under clinical investigations for its utility in type 2 diabetes and non-alcoholic steatohepatitis^55^. The ability of FGF21-receptor agonists to affect VAT inflammation might be a key mechanism by which these therapeutic proteins induce weight loss and improve metabolic defects in obese humans that do not possess much brown adipose tissues.

The adaptive tissue remodeling that we observed in the sympathoactivated obese VAT is also reminiscent of the wound healing process in which pro-inflammatory, pro-fibrotic, and anti-inflammatory macrophages play critical roles^56^. The roles of the SNS in tissue regeneration and injury repair have also been described previously^57,58^. Through ATM gene expression profiling, we observed an induction of GDF15, a recently described GFRAL/RET ligand, in ATM2 in the sympatho-stimulated VAT. The expression of GDF15 is also at the site of tissue injury and promotes tissue repairs^59–61^. GDF15 is also highly expressed in malignant tumor and promote tumorigenesis and immunosuppression, consistent its function in regulating a neuroimmune-modulatory circuit^39,62^. The contributions of SNS in tissue injury repair, tumorigenesis, and immunosuppression have also been documented previously^57,58^. The roles of ATM in controlling catecholamine catabolism and tissue innervation have also recently been reported^63–65^. Thus, GDF15 might act as a macrophage-derived neurotrophic factor that regulates the nervous system in remodeling tissues in general. It is unclear whether the receptors for GDF15 is expressed in SNS or other stromal cells, thus further study is required to test the role of GDF15 in remodeling tissues.

## Methods

### Mouse models

All animal studies were conducted in accordance with the Guide for the Care and Use of Laboratory Animals, published by the National Institutes of Health (NIH) (NIH Publication 8523, revised 1985). The Institutional Animal Care and Use Committee (IACUC) at Genentech reviewed and approved all animal protocols. Male C57BL/6 mice on standard chow (#000664) or high-fat diet (HFD, #380050) were from The Jackson Laboratory. All the mice were maintained in a pathogen-free animal facility under standard 12 h light/12 h dark cycle with access to standard chow (Labdiet 5010) or HFD (Harlan Teklad TD.06414, 58.4% calories from fat) and water ad libitum at 21 °C unless indicated otherwise. DIO mice were placed on HFD at 6 weeks of age and for at least 12 weeks until they reach 38–45 g of body weight before cold exposure or antibody treatment. Mice were randomized into groups based on their body weight. For cold exposure studies, before exposure to a temperature of 4 °C, mice were acclimated at 18 °C for 5 or 7 days before reducing the temperature down to 4 °C, per recommendation by Genentech IACUC. Serum total cholesterol, triglyceride, and alanine aminotransferase (ALT) levels were determined by standard photometric assays on AU480 analyzer (Beckman Coulter). Blood glucose levels were determined using a Contour glucose meter (Bayer).

CSF1 and IL4 concentrations in adipose tissues were measured with the Bio-Plex cytokine assay (Bio-Rad Laboratories). Briefly, tissue was homogenized in RIPA buffer and diluted in 1% BSA PBS to 1:16. The supernatant was incubated with magnetic beads and detection antibodies sequentially at RT. After incubation, streptavidin-PE was added to the samples and analyzed with the Bio-Plex 200 system (Bio-Rad Laboratories).

### Flow cytometry and cell sorting

To SVF cells for flow cytometry, adipose tissues were harvested, shredded with scissors and enzymatically digested, as previously described^66^. Briefly, tissues were incubated in digestion enzymes for 15 min at 37 °C with vigorous agitation, non-adipocytes were removed and placed in fresh RPMI with 5% FBS without digestion enzymes. This was repeated twice. Cells were pelleted and resuspended in ACK lysis buffer to lyse red blood cells, washed, filtered through a final 30 μM filter and counted on the ViCell automated cell counter. To identify the macrophage population, a gating strategy similar to a previously described was used^67^. Specifically, we utilized Gr1, Fcer1, and Siglec-f as a Dump channel to exclude out neutrophil, basophil, and eosinophil, and F4/80^+^/CD11b^+^/Gr1^−^ /Fcer1^−^/Siglec-f^−^ cells were defined as total ATM. For FACS analysis, after Fc-blocking (anti-mouse CD16/CD32, BD Biosciences), various cell-surface markers were detected with fluorochrome-conjugated antibodies. Description of antibodies is in Supplemental Information (Supplemental Table 1). All antibodies were used at 1:200 dilution, except for Lyve1 used at 1:50, and cells were stained for 40–60 min on ice. Data were acquired using an LSR II or Fortessa (BD Biosciences) and analyzed using FlowJo software (TreeStar version 10.1r5). Doublets were excluded before gating on live cells. For cell sorting, cells were prepared as described above and sorted on a Fusion or Aria (BD Biosciences).

For magnetic-activated cell sorting (MACS), cells were incubated with anti-CD45 and anti-CD31 magnetically labeled beads (Miltenyi Biotec). CD45 and CD31 positive cells were then depleted using LS columns per the manufacturer’s instruction. The resulting cells were plated for *in vitro* experiments.

### Histological analysis

Adipose tissues were fixed in 10% formalin for 24 h, washed with PBS three times and stored in 70% ethanol at 4 °C. Fixed tissues were embedded in paraffin and sectioned (7 μm). After deparaffinization in xylene and rehydration through graded alcohols to distilled water, slides were stained with hematoxylin and eosin (H&E) or Picro Sirius Red (ab150681, Abcam) and imaged with bright-field microscopy or polarized light microscopy.

For immunofluorescence (IF), slides were pretreated with antigen retrieval solution for 20 min at 99 °C, followed by 3% hydrogen peroxide in PBS. Sections were then blocked in PBS containing 3% BSA and 10% donkey serum for 1 h at RT. After stained with primary antibodies (supplemental information), slides were washed (0.05 M Tris, 0.15 M NaCl, 0.05% Tween20) and incubated with a cocktail of secondary antibodies (Alexa Flour, ThermoFisher) for 1 h at RT. Slides were washed and mounted with either VECTASHIELD or Prolong Gold mounting medium. For Arg1 (ab124917, Abcam) and F4/80 (MCA497G, Bio-Rad Laboratories) staining, the process required additional steps: After primary antibody staining, sections were incubated with biotinylated labeled secondary antibody for 60 min at RT. After washing, sections were incubated with diluted VECTASTAIN ABC Elite Kits (Vector, CA) for 30 min, washed, and subjected to signal amplification with Tyramide Signal Amplification (TSA) kits (Life Technologies) for 5–20 min at RT. In order to detect hyaluronan, slides were blocked in PBS-Tween containing 1% BSA, incubated with biotin-conjugated hyaluronan binding protein (HABP) (EMD/Millipore #385991) (1:100) overnight at 4 °C, washed and incubated with Alexa Fluor 594 conjugated streptavidin (1:500) for 1 h at RT. Slides were washed and mounted. All slides were imaged by LEICA SPE confocal microscope or Zeiss Axio microscope.

### Western blot analysis

Adipose tissues were homogenized in standard RIPA buffer with cOmplete™ Protease Inhibitor Cocktail Tablets (Roche) and/or PhosSTOP (Roche), centrifuged at 13,000 × *g* for 20 min at 4 °C to remove the fat layer. Protein extracts were separated on 4–12% NuPAGE gels (Invitrogen) and blotted onto nitrocellulose membranes (iBlot, Invitrogen). Membranes were blocked and incubated with primary antibodies (supplemental information). After washing, membranes were incubated with HRP-linked secondary antibodies (Cell Signaling). Signals were detected using the ECL Prime Western blotting detection reagent and visualized on Amersham Biosciences Hyperfilm ECL. Signal intensity was quantified by ImageJ, and the correlation was calculated by Prism 7. The raw image data are provided in Supplemental Figures 6–8.

### RNA preparation and quantitative PCR (qPCR)

Total RNA was extracted from tissues or cells using QIAzol Lysis Reagent (QIAGEN) along with RNeasy kits (QIAGEN). For qPCR analysis, RNA was reverse transcribed using the iScript™ cDNA Synthesis Kit (Bio-Rad Laboratories). cDNA was analyzed with specific primer sets (supplemental information) in SYBR® GreenER™ qPCR SuperMix for ABI PRISM (Thermo Fisher Scientific) by using QuantStudio 5 Real-Time PCR System (Thermo Fisher Scientific). Relative mRNA expression was determined by normalizing by *Tbp* (TATA-box binding protein) or *Rpl13a* (ribosomal protein L13a) levels as relative mRNA expression.

### Genome-wide RNA-Seq gene expression analysis

For RNA-Seq analysis, FACS sorted cells from 5 mice were pooled as one sample for ATM1 and ATM2, and cells from 3 mice were pooled as one sample for AP. 3 total RNA samples per group for each cell type/condition were prepared using RNeasy Kit (Qiagen). RNA-Seq libraries were prepared using TruSeq RNA sample preparation kit (Illumina). The libraries were sequenced on HiSeq 2500 and produced on average 48 million single-end 50 bp reads per sample. RNA-Seq reads were aligned to the mouse genome GRCm38 using GSNAP^68^. Expression counts per gene were obtained by counting the number of reads aligned uniquely to each gene locus as defined by NCBI and Ensembl gene annotations and RefSeq mRNA sequences. Differential gene expression analysis was performed using edgeR (Version 3.18.1, Bioconductor)^69^ and genes with FDR corrected p-values of 0.0001 or less were considered significant and are included in the supplemental tables. DESeq (Version 1.28.0, Bioconductor)^70^ was used to compute the variance stabilized expression values for plotting the expression heatmaps. tSNE values were computed using the Rtsne library (https://github.com/jkrijthe/Rtsne). The comparison of expression fold changes of cold versus warm and bFKB1 versus warm was calculated using edgeR for differential expression analysis. Gene set enrichment analysis was performed using the GSEA analysis tool (Version 3.0)^71^. For gene ontology (GO) enrichment analysis, differentially expressed genes were selected by FDR < 10–4, and |log_2_(fold change)| > 0.7 for M1 genes, |log_2_(fold change)| > 0.6 for AP genes, |log_2_(fold change)| > 0.3 for ATM2 genes, and were subjected for gene ontology and pathway analysis using g:Profiler with default settings (http://biit.cs.ut.ee/gprofiler/index.cgi). To generate heatmaps of differentially expressed genes from selected enriched GO terms, RNA-Seq data of selected genes were hierarchically clustered. Data (log_2_(RPKM)) were first standardized along the rows such that the mean is 0 and the standard deviation is 1. The genes in Fig. 6J were selected by requiring them to have an average RPKM more than 5 in bFKB1-treated samples and have a fold expression change larger than 1.86 or smaller than 0.53 when compared to the Warm/Control group. The values plotted in the heatmap are row Z-score scaled log_2_(RPKM). Euclidean distance and Ward linkage were used for clustering (MATLAB 9.1.0). RNA-Seq data is available at the NCBI (GSE112396).

### Recombinant proteins

Production of bFKB1 was previously described^49^. Mature human GDF15 protein (A197-I308) was expressed from a CHO stable pool and purified using a His-tag inserted downstream of the pro-domain cleavage site (-RRRARGHHHHHH-). In addition, mature hGDF15 was expressed in HEK293T cells as a fusion to human IgG1 Fc fragment using a strategy similar to a previously described^72^. Recombinant BMP2 and GDNF were from R&D Systems.

### Cell culture

MACS-sorted fibroblast-like progenitor cells were plated and cultured in DMEM containing 10% calf serum overnight. Flow cytometry showed that 70% of the MACS-sorted cells were CD45^-^/PDGFRα^+^/Sca1^+^/CD29^+^ AP (data not shown). Cells were cultured overnight, washed with serum-free DMEM three times before ligand was added for phospho-Smad analysis.

For qPCR analysis of differentiated AP, FACS sorted AP were plated and cultured in DMEM containing 10% calf serum until they reached confluence. Cell were then cultured in growth media containing insulin (1 μM), dexamethasone (5 μM), triiodothyronine (2 nM), rosiglitazone (15 μM), IBMX (75 μM), indomethacin (125 μM), and a BMP2/GDF15 ligand for 4 days, then in growth media containing insulin (1 μM) dexamethasone (5 μM), triiodothyronine (2 nM), and a BMP2/GDF15 ligand for 10 days. Cells were harvest for qPCR gene expression analysis.

For GAL-ELK1-based luciferase assay, HEK293T cells were transiently transfected with expression vectors encoding human RET (RET51 isoform) and/or a GFRA coreceptor under the CMV-promoter (Genentech), Renilla luciferase (pRL-SV40, Promega), GAL-ELK1 transcriptional activator fusion (pFA2-ELK1, Agilent), and a firefly luciferase reporter driven by GAL4 binding sites (pFR-luc, Agilent), using FuGENE HD Transfection Reagent (Promega). On the next day, the transfected cells were cultured for an additional 6–8 h in serum-free DMEM-based media containing appropriate protein ligands at various concentrations. The cellular luciferase activity was determined using Dual-Glo Luciferase Assay System (Promega) and EnVision Multilabel Reader (PerkinElmer). Firefly luciferase activity was normalized to the co-expressed Renilla luciferase activity. All the luciferase assays were run in triplicate, and the results are shown as means ± SEM.

### Quantification and statistical analysis

One-way ANOVA with post-hoc Dunnett’s test or unpaired Student’s *t*-test (two-tailed) was used for statistical analysis to compare treatment groups. A *P* value of <0.05 was considered statistically significant. “ns” stands for no significance. All the values were presented as means ± SEM.

## Supplementary information

Supplemental Information

## Data Availability

All data generated or analysed during this study are included in this published article and its Supplementary Information file.
