# Supplementary material for: Adaptive adipose tissue stromal plasticity in response to cold stress and antibody-based metabolic therapy
Source: Sci Rep. 2019 Jun 20;9:8833. doi: 10.1038/s41598-019-45354-1 (PMC6586812; doi:10.1038/s41598-019-45354-1)
Supplement: Supplementary file 1 — Supplemental Information [file 41598_2019_45354_MOESM1_ESM.pdf]

Supplemental Information

Adaptive adipose tissue stromal plasticity in response to cold stress and antibody-based metabolic therapy

Joshua C. Chang, Steffen Durinck, Mark Z. Chen, Nadia Martinez-Martin, Jingli A. Zhang, Isabelle Lehoux, Hong Li, May Lin, Jiansheng Wu, Travis W. Bainbridge, James A. Ernst, Sree R. Ramani, Sairupa Paduchuri, Lance Kates, Margaret Solon, Matthew B. Buechler, Alessandra Castiglioni, Minh Thai, Beatrice Breart, Zora Modrusan, Andrew S. Peterson, Shannon J. Turley, Junichiro Sonoda

Fig. S1

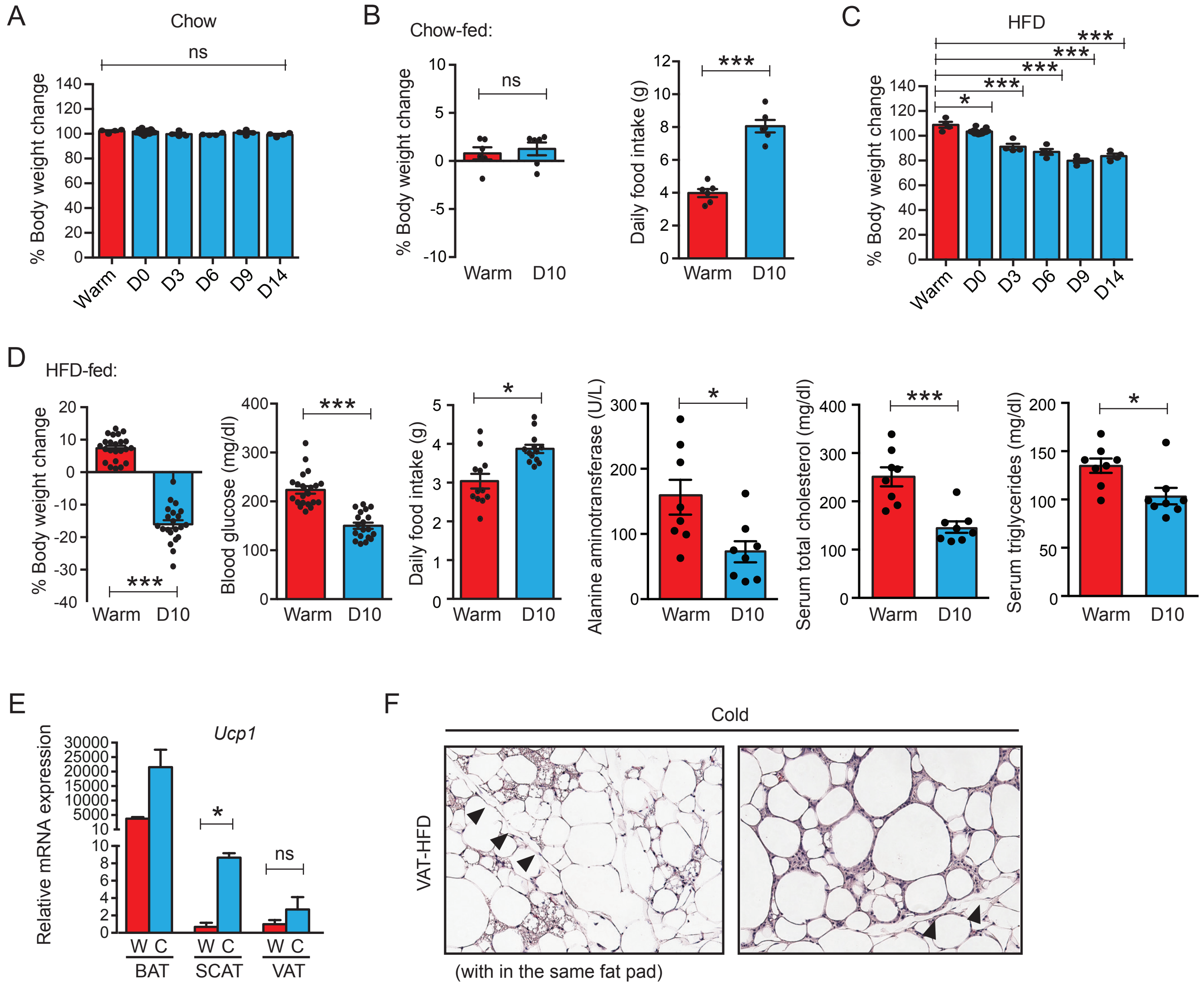

A
Chow
ns
% Body weight change
Warm D0 D3 D6 D9 D14
B
Chow-fed:
ns
% Body weight change
Warm D10
***
Daily food intake (g)
Warm D10
C
HFD
*
***
% Body weight change
Warm D0 D3 D6 D9 D14
D
HFD-fed:
% Body weight change
***
Warm D10
Blood glucose (mg/dl)
Daily food intake (g)
Alanine aminotransferase (U/L)
Serum total cholesterol (mg/dl)
Serum triglycerides (mg/dl)
E
Ucp1
Relative mRNA expression
W C
BAT
SCAT
VAT
ns
F
Cold
VAT-HFD
(with in the same fat pad)

Supplemental Figure S1. Cold exposure induces weight loss and browning in obese mice. C57BL/6 mice on either **(A-B)** normal chow (10% kcal fat) or **(C-F)** HFD (60% kcal fat) were maintained at thermoneutrality (30°C, Warm) or exposed to 4°C (Cold) after adaptation at 18°C. (**A**) Body weight change % (from day -5) after exposed to 4°C cold for the indicated time, or control Warm mice maintained under thermoneutrality until day 14 (red) (N=4 mice per group). The starting body weights were 29.8 ± 0.2 g. **(B)** Body weight changes and food intake of chow-fed mice during 4°C cold exposure for 10 days. (N=6 mice per group). **(C)** Body weight changes % (from day -5) of HFD-fed DIO mice after exposed to 4°C cold for the indicated time, or control Warm mice maintained under thermoneutrality until day 14 (red) (N=4 mice per group). The starting body weights were 46.5 ± 0.7 g. **(D)** Body weight changes, blood glucose (N≥18 mice per group), food intake (N=12 mice per group), serum total alanine aminotransferase, cholesterol, and triglycerides (N=8 mice per group) in DIO mice after 4°C cold exposure for 10 days. **(E)** qPCR analysis of *Ucp1* mRNA expression in BAT (N=3), SCAT, and VAT (N=5~6) in cold exposed (10 days) DIO mice. Statistical analysis was conducted without considering BAT samples. **(F)** H&E staining of cold exposed HFD-fed VAT. Both images are from the same fat pad. Triangles indicate fibrous septa. *P<0.05, **P<0.01, ***P<0.001 by one-way ANOVA (A and C) or by student t-test (B, D and E). Data are shown as mean ± SEM.

Fig. S2

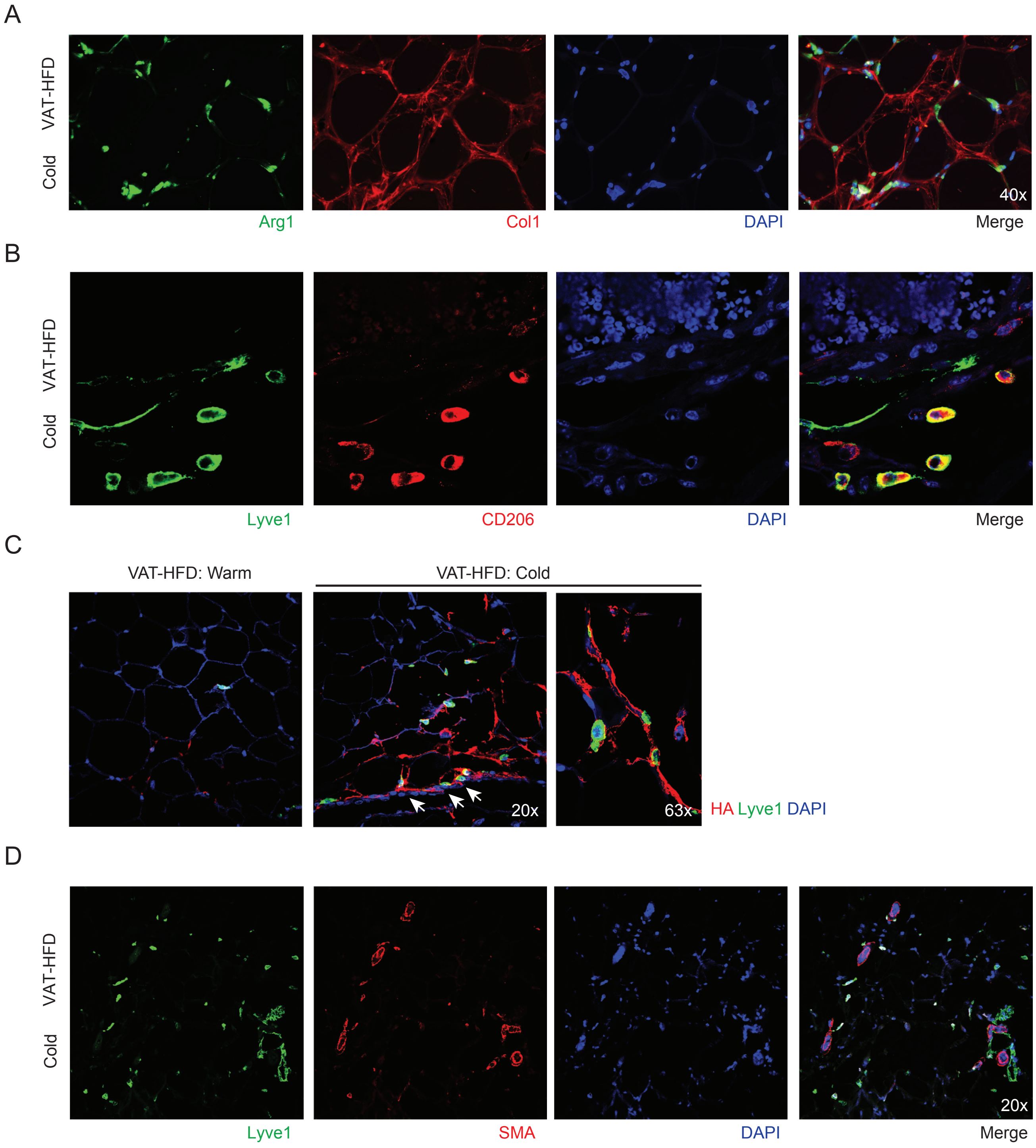
A
VAT-HFD Cold
40x
Arg1
Col1
DAPI
Merge
B
VAT-HFD Cold
Lyve1
CD206
DAPI
Merge
C
VAT-HFD: Warm
VAT-HFD: Cold
20x
63x
HA Lyve1 DAPI
D
VAT-HFD Cold
20x
Lyve1
SMA
DAPI
Merge

Supplemental Figure S2. Cold exposure induces VAT remolding in obese mice. HFD-fed mice were maintained at 30°C (Warm) or exposed to 4°C for 10 days (Cold). **(A)** The same images as Figure 2B split into single channels. **(B)** The same images as Figure 2D split into single channels. **(C)** HA (red) and Lyve1 (green) IF. **(D)** The same images as Figure 2F split into single channels. DAPI (blue) stains cell nuclei in all IF.

Fig. S3

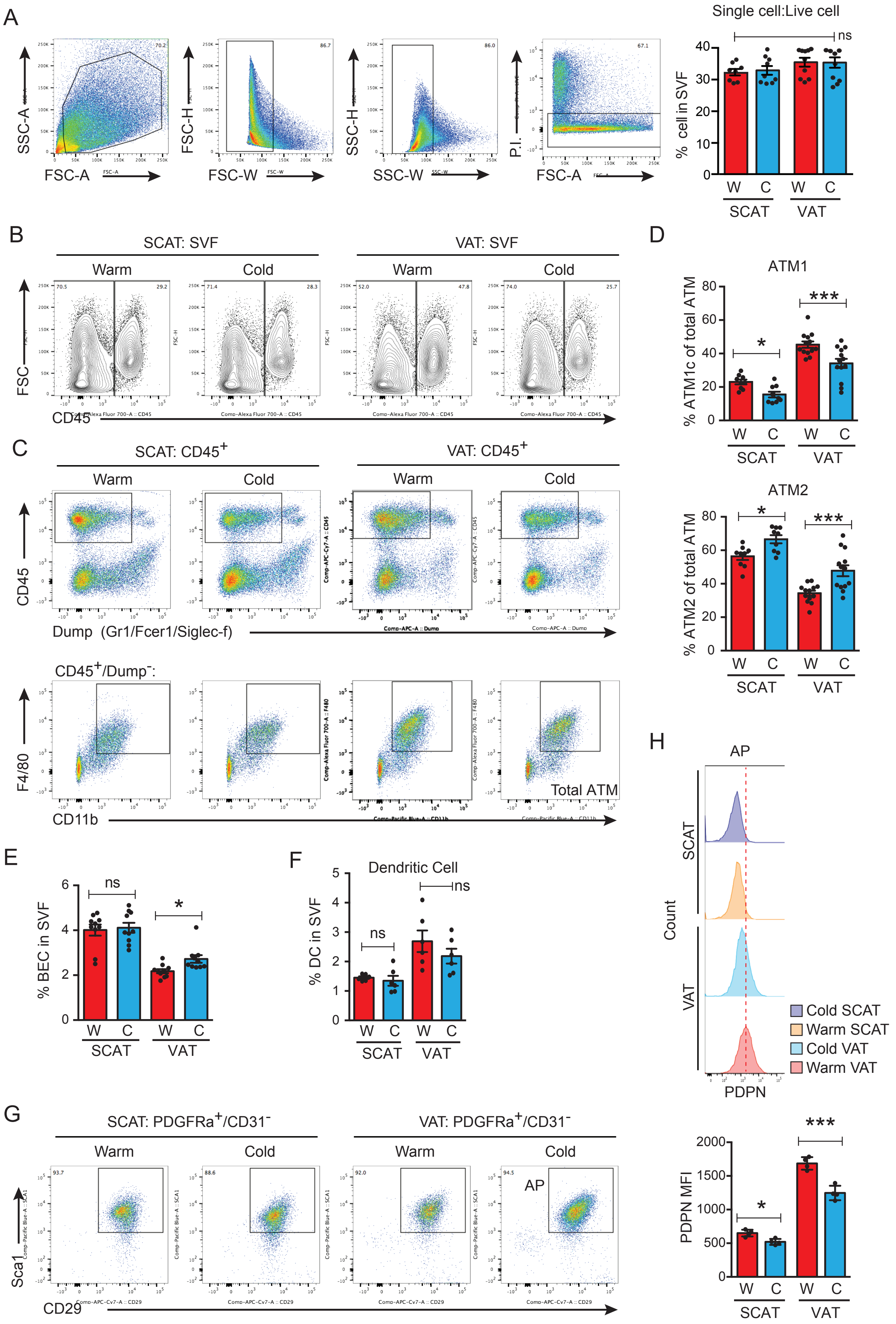

A
Single cell:Live cell
ns
% cell in SVF
SSC-A
FSC-A
FSC-H
FSC-W
SSC-H
SSC-W
P.I.
W C W C
SCAT VAT
B
SCAT: SVF
VAT: SVF
Warm Cold Warm Cold
FSC
CD45
C
SCAT: CD45+
VAT: CD45+
Warm Cold Warm Cold
CD45
Dump (Gr1/Fcer1/Siglec-f)
CD45+/Dump-:
F4/80
CD11b
Total ATM
D
ATM1
% ATM1c of total ATM
ATM2
% ATM2 of total ATM
E
% BEC in SVF
F
Dendritic Cell
% DC in SVF
G
SCAT: PDGFRa+/CD31-
VAT: PDGFRa+/CD31-
Warm Cold Warm Cold
AP
Sca1
CD29
H
AP
SCAT
VAT
Count
PDPN
Cold SCAT
Warm SCAT
Cold VAT
Warm VAT
PDPN MFI

Supplemental Figure S3. Cold exposure recruits ATM2. HFD-fed mice were maintained at 30°C (Warm) or exposed to 4°C for 10 days (Cold). Flow cytometry analysis of SVF from both SCAT and VAT are shown. **(A)** The gating strategy of live cells in adipose tissue SVF. Frequencies of live cells in SVF at right. **(B)** Representative flow cytometry plots showing $CD45^+$ or $CD45^-$ cells in live SVF. **(C)** Representative flow cytometry plots showing $CD45^+/F4/80^+/CD11b^+/Dump^-$ ATM in total live SVF. **(D)** Frequencies of $CD11c^+/CD206^-$ ATM1 and $CD11c^-/CD206^+$ ATM2 in total $CD45^+/F4/80^+/CD11b^+/Dump^-$ ATM. **(E)** Frequencies of $CD31^+/PDPN^-/CD45^-$ BEC in total SVF. **(F)** Frequencies of $CD45^+/Dump^-/F4/80^-/CD11c^+$ dendritic cells in total SVF. **(G)** Representative flow cytometry plots showing $CD45^-/CD31^-/PDGFR\alpha^+/Sca1^+/CD29^+$ AP. **(H)** Flow cytometry histogram showing PDPN expression level in AP (left) and the quantification of the PDPN MFI (right). PDPN is an inflammation marker. $^*P<0.05$, $^{**}P<0.01$, $^{***}P<0.001$ by student t-test. Data are shown as mean ± SEM.

Fig. S4

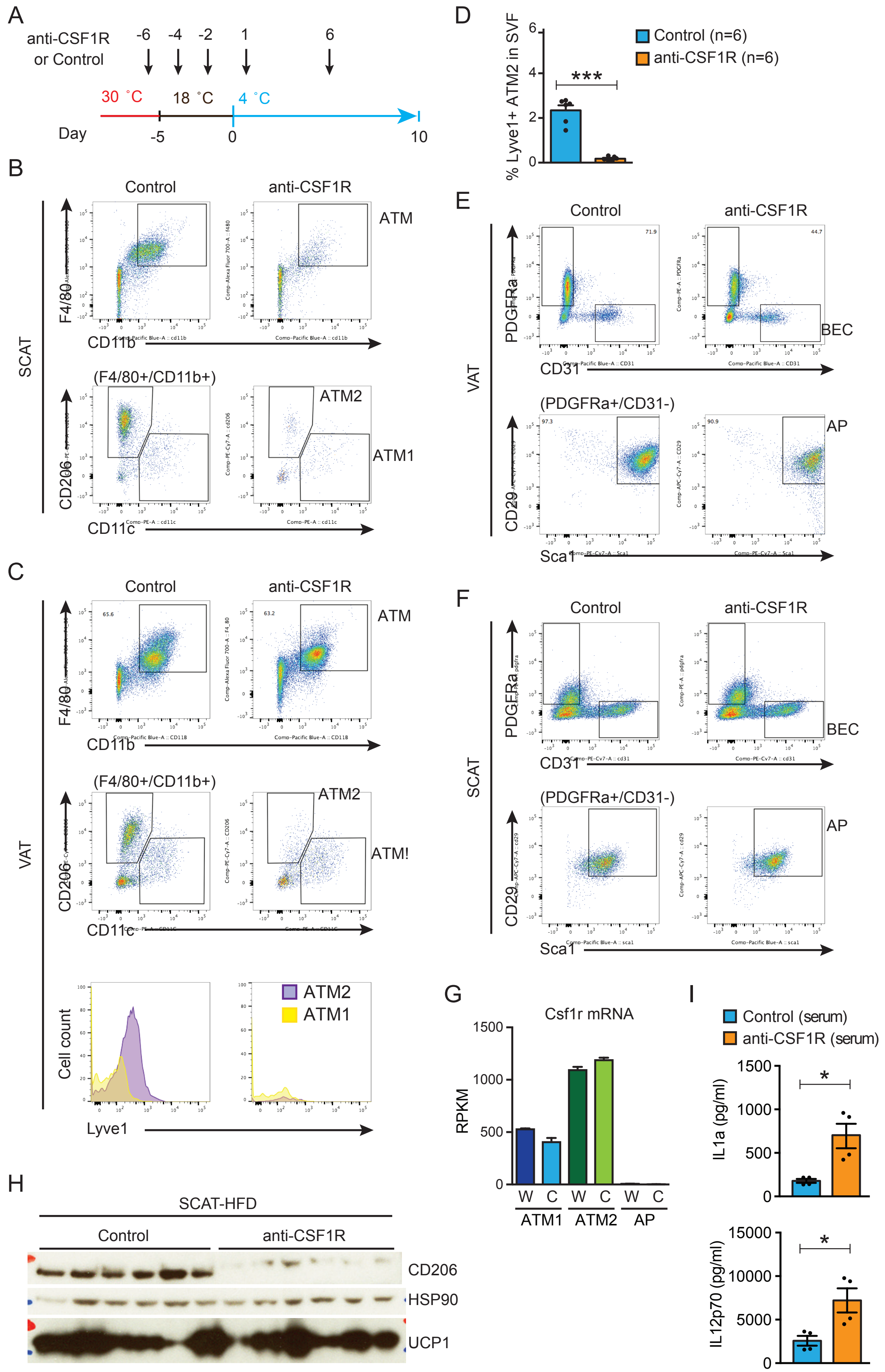
A
anti-CSF1R
or Control
-6
-4
-2
1
6
30 ˚C
18 ˚C
4 ˚C
Day
-5
0
10
B
Control
anti-CSF1R
ATM
SCAT
F4/80
CD11b
(F4/80+/CD11b+)
ATM2
ATM1
CD206
CD11c
C
VAT
ATM!
Cell count
Lyve1
D
% Lyve1+ ATM2 in SVF
***
Control (n=6)
anti-CSF1R (n=6)
E
PDGFRa
CD31
BEC
(PDGFRa+/CD31-)
AP
CD29
Sca1
F
G
Csf1r mRNA
RPKM
W
C
AP
H
SCAT-HFD
CD206
HSP90
UCP1
I
Control (serum)
anti-CSF1R (serum)
IL1a (pg/ml)
IL12p70 (pg/ml)
*

Supplemental Figure S4. CSF1R signaling is required for cold-induced recruitment of ATM2. (A) The design for the anti-CSF1R experiment. HFD-fed C57BL/6 male mice maintained at thermoneutrality were i.p. dosed with 40 mg/kg anti-CSF1R antibody or isotype control at indicated times (arrows). All the mice were exposed to 4°C for 10 days after acclimation for 5 days at 18 °C. **(B)** Representative flow cytometry plots showing total ATM (top), ATM1 and ATM2 (bottom) in SCAT with or without anti-CSF1R treatment. **(C)** Representative flow cytometry plots showing total ATM (top), ATM1 and ATM2 (middle), and Lyve1 histograms in ATM1 and ATM2 populations (bottom) in VAT, with or without anti-CSF1R treatment. **(D)** Frequencies of Lyve1^{+} ATM2 in total SVF with or without anti-CSF1R treatment. **(E)** Representative FACS plots showing AP in VAT with or without anti-CSF1R treatment. **(F)** Representative FACS plots showing AP in SCAT with or without anti-CSF1R treatment. **(G)** *Csf1r* mRNA expression in ATM1, ATM2, and AP in warm or cold-exposed VAT by RNA-seq (N=3). **(H)** Western blot analysis for CD206 and UCP1 expression in obese SCAT with or without anti-CSF1R treatment. HSP90 serves as loading control. (I) Cytokine concentrations in serum. **P*<0.05, ****P*<0.001 by student t-test. Data are shown as mean ± SEM.

Fig. S5

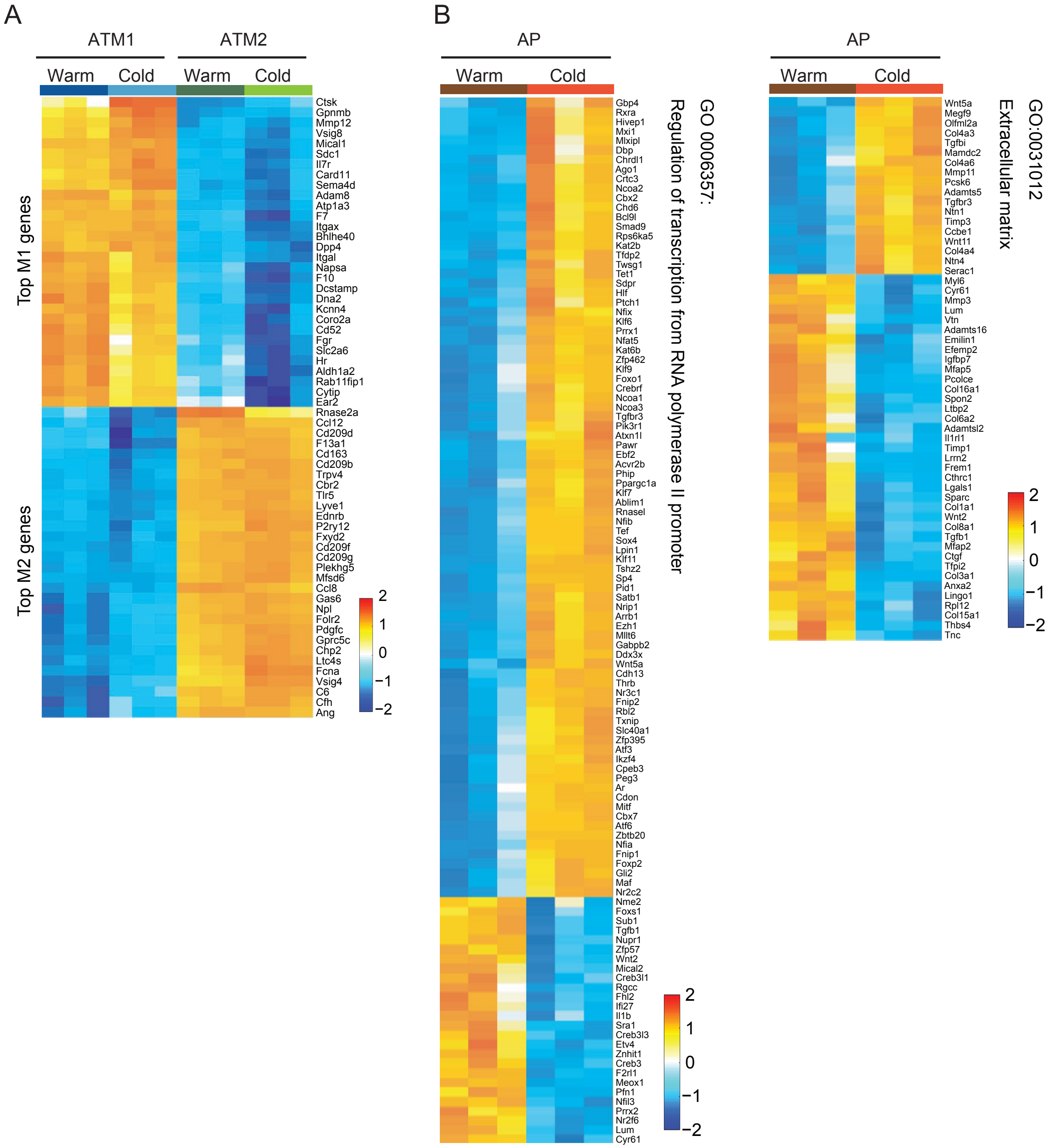
A
ATM1
ATM2
Warm
Cold
Warm
Cold
Top M1 genes
Top M2 genes
Ctsk
Gpnmb
Mmp12
Vsig8
Mical1
Sdc1
Il7r
Card11
Sema4d
Adam8
Atp1a3
F7
Itgax
Bhlhe40
Dpp4
Itgal
Napsa
F10
Dcstamp
Dna2
Kcnn4
Coro2a
Cd52
Fgr
Slc2a6
Hr
Aldh1a2
Rab11fip1
Cytip
Ear2
Rnase2a
Ccl12
Cd209d
F13a1
Cd163
Cd209b
Trpv4
Cbr2
Tlr5
Lyve1
Ednrb
P2ry12
Fxyd2
Cd209f
Cd209g
Plekhg5
Mfsd6
Ccl8
Gas6
Npl
Folr2
Pdgfc
Gprc5c
Chp2
Ltc4s
Fcna
Vsig4
C6
Cfh
Ang
2
1
0
−1
−2
B
AP
Warm
Cold
GO 0006357:
Regulation of transcription from RNA polymerase II promoter
Gbp4
Rxra
Hivep1
Mxi1
Mlxipl
Dbp
Chrdl1
Ago1
Crtc3
Ncoa2
Cbx2
Chd6
Bcl9l
Smad9
Rps6ka5
Kat2b
Tfdp2
Twsg1
Tet1
Sdpr
Hlf
Ptch1
Nfix
Klf6
Prrx1
Nfat5
Kat6b
Zfp462
Klf9
Foxo1
Crebrf
Ncoa1
Ncoa3
Tgfbr3
Pik3r1
Atxn1l
Pawr
Ebf2
Acvr2b
Phip
Ppargc1a
Klf7
Ablim1
Rnasel
Nfib
Tef
Sox4
Lpin1
Klf11
Tshz2
Sp4
Pld1
Satb1
Nrip1
Arrb1
Ezh1
Mllt6
Gabpb2
Ddx3x
Wnt5a
Cdh13
Thrb
Nr3c1
Fnip2
Rbl2
Txnip
Slc40a1
Zfp395
Atf3
Ikzf4
Cpeb3
Peg3
Ar
Cdon
Mitf
Cbx7
Atf6
Zbtb20
Nfia
Fnip1
Foxp2
Gli2
Maf
Nr2c2
Nme2
Foxs1
Sub1
Tgfb1
Nupr1
Zfp57
Wnt2
Mical2
Creb3l1
Rgcc
Fhl2
Ifi27
Il1b
Sra1
Creb3l3
Etv4
Znhit1
Creb3
F2rl1
Meox1
Pfn1
Nfil3
Prrx2
Nr2f6
Lum
Cyr61
2
1
0
−1
−2
AP
Warm
Cold
GO:0031012
Extracellular matrix
Wnt5a
Megf9
Olfml2a
Col4a3
Tgfbi
Mamdc2
Col4a6
Mmp11
Pcsk6
Adamts5
Tgfbr3
Ntn1
Timp3
Ccbe1
Wnt11
Col4a4
Ntn4
Serac1
Myl6
Cyr61
Mmp3
Lum
Vtn
Adamts16
Emilin1
Efemp2
Igfbp7
Mfap5
Pcolce
Col16a1
Spon2
Ltbp2
Col6a2
Adamtsl2
Il1rl1
Timp1
Lrrn2
Frem1
Cthrc1
Lgals1
Sparc
Col1a1
Wnt2
Col8a1
Tgfb1
Mfap2
Ctgf
Tfpi2
Col3a1
Anxa2
Lingo1
Rpl12
Col15a1
Thbs4
Tnc
2
1
0
−1
−2

Supplemental Figure S5. Heatmap showing ATM and AP gene expression in warm and cold conditions. (**A**) Heatmap showing top 20 ATM1 and ATM2 gene and their expression in warm and cold conditions. **(B)** Heatmap showing AP expression of various genes in indicated GO pathways. For heatmaps, expression data (log_2(RPKM)) are standardized along each gene (row), and color scale corresponds to the relative expression level.

Fig. S6

A

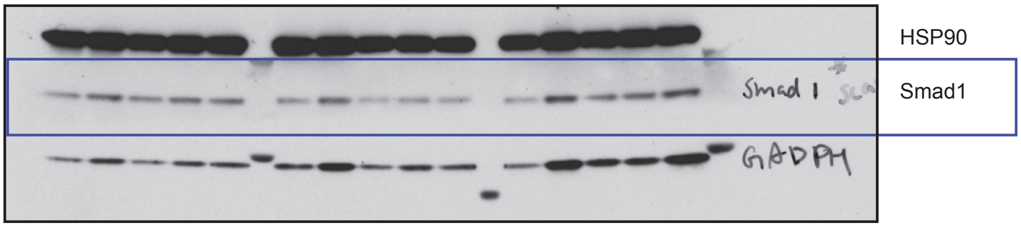
HSP90
Smad 1
Smad1
GADPH

B

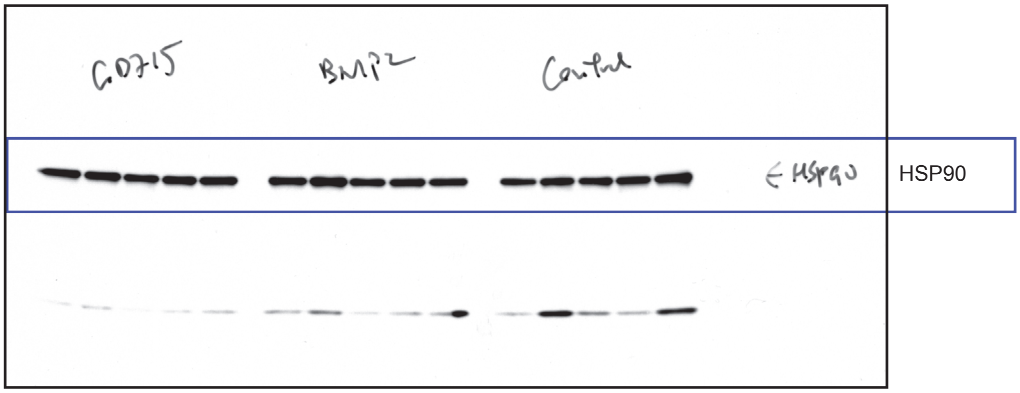
GDF15
BMP2
Control
HSP90
HSP90

C

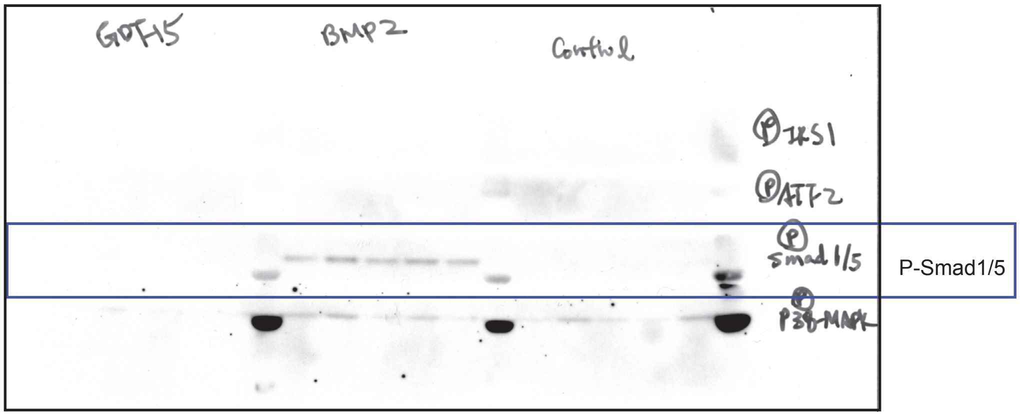
GDF15
BMP2
Control
P-Smad1/5

Supplemental Figure S6. Scanned raw images for the western blot images shown in Figure 5C. Nitrocellulose membranes were cut into stripes for different antibody staining. (A) and (B) are the same blot with different exposure time. (C) is a different blot from (A) and (B), but from a gel loaded with the same amount of proteins, with the same order, and ran on the same time in the same gel tank. Blue boxes indicate the cropped images shown in Figure 5C. In Figure 5C, the images are flipped horizontally to show control samples on the left.

Fig. S7

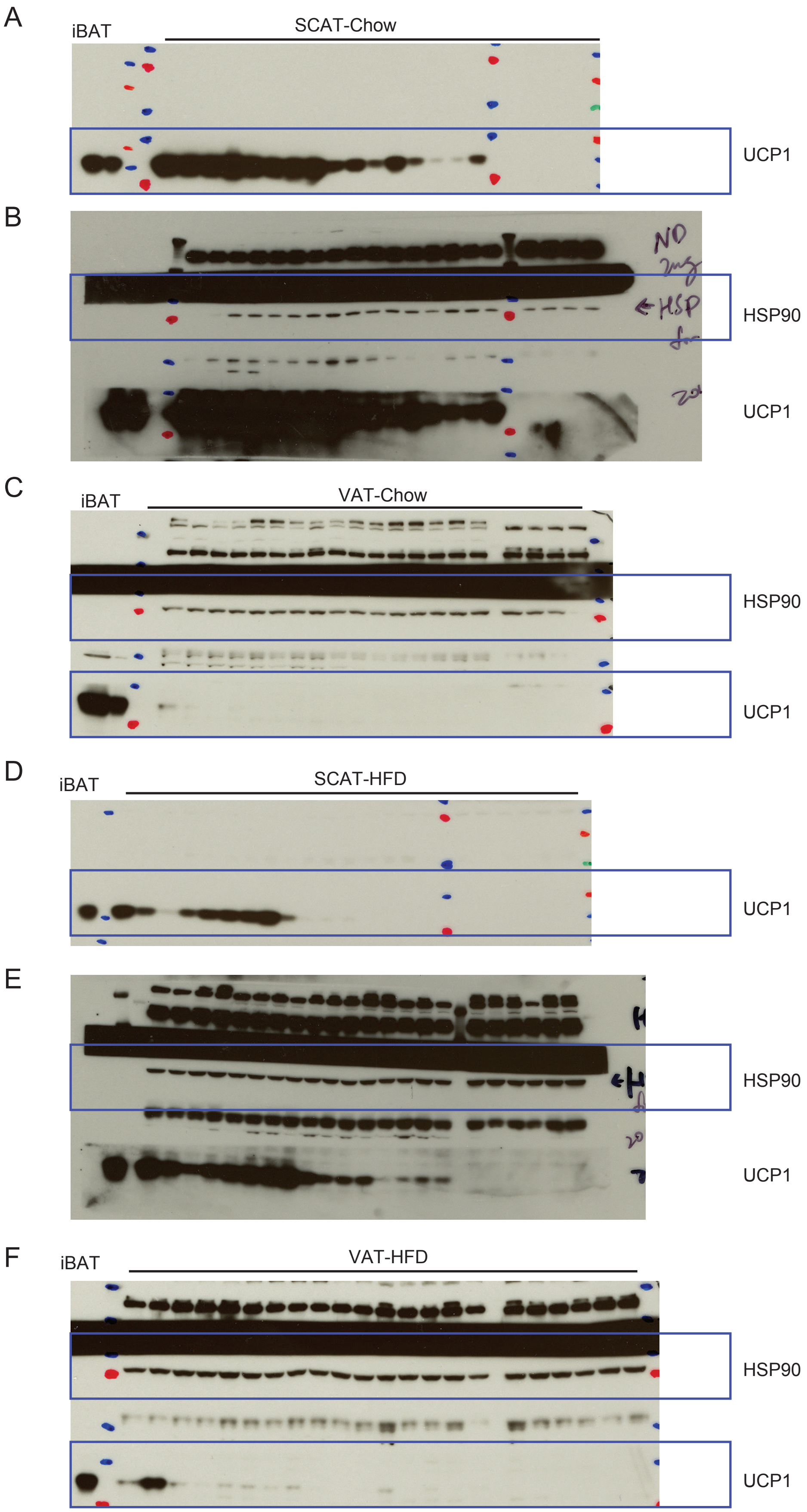
A
iBAT
SCAT-Chow
UCP1
B
HSP90
UCP1
C
iBAT
VAT-Chow
HSP90
UCP1
D
iBAT
SCAT-HFD
UCP1
E
HSP90
UCP1
F
iBAT
VAT-HFD
HSP90
UCP1

Supplemental Figure S7. Scanned raw images for the western blot images shown in Figure 1D and F. Nitrocellulose membranes were cut in stripes for different antibody staining. (A) and (B) are the same blots with different exposure time. (C) HSP90 and UCP1 expressions were shown in the same blot. (D) and (E) are the same blots with different exposure time. (F) HSP90 and UCP1 expressions were shown in the same blot. Blue boxes indicate the cropped images shown in Figure 1D and F. In Figure 1D and F, the images are flipped horizontally to show control samples on the left.

Fig. S8

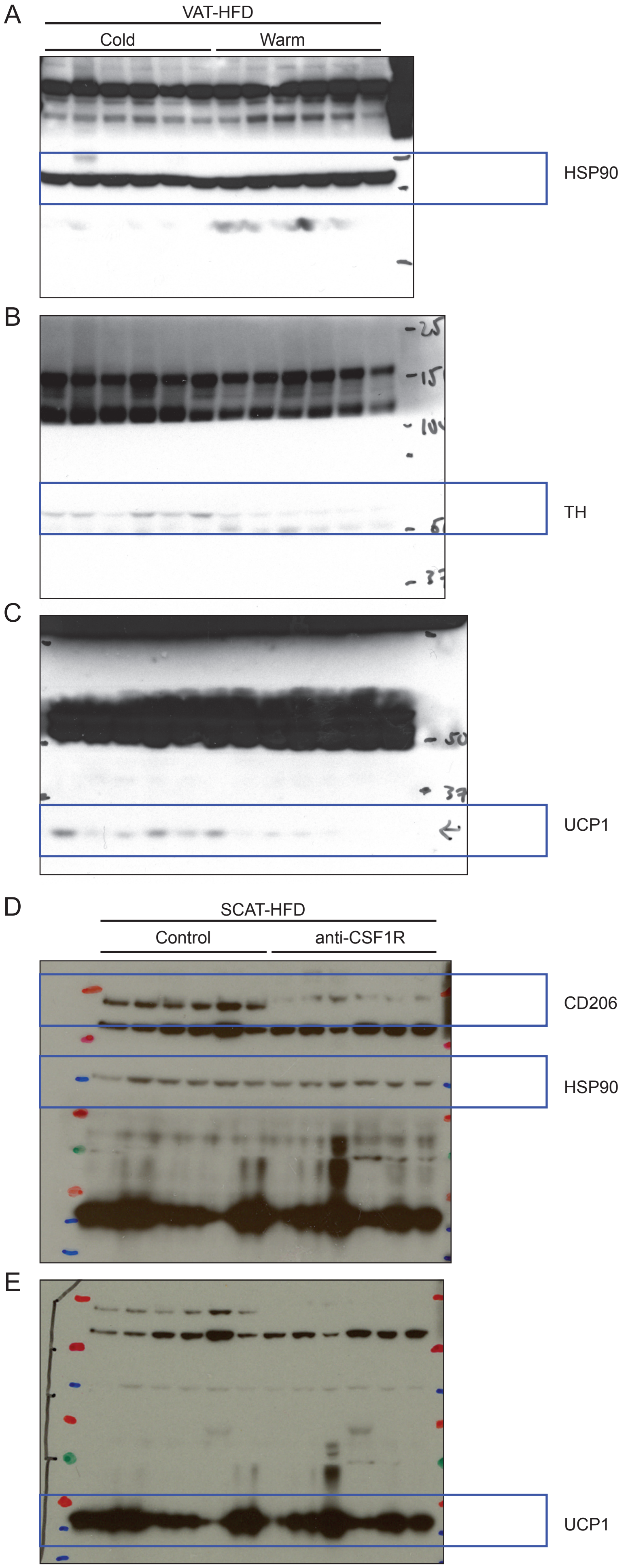
A
VAT-HFD
Cold
Warm
HSP90
B
TH
C
UCP1
D
SCAT-HFD
Control
anti-CSF1R
CD206
HSP90
E
UCP1

Supplemental Figure S8. Scanned raw images for the western blot images shown in Figure 2I and Supplemental Figure S4H. Nitrocellulose membranes were cut in stripes for different antibody staining. (A) gel was processed and loaded the same amount of proteins as (B) and (C). (B) and (C) are the same blots with different exposure time. (A) HSP90, (B) TH, and (C) UCP1 expressions were shown. (D) and (E) are the same blots with different exposure time. (D) HSP90 and CD206, and (E) UCP1 expressions were shown in the same blot. Blue boxes indicate the cropped images shown in Figure 2I and Supplemental Figure S4H. In Figure 2I, the images are flipped horizontally to show control samples on the left.

Fig. S9

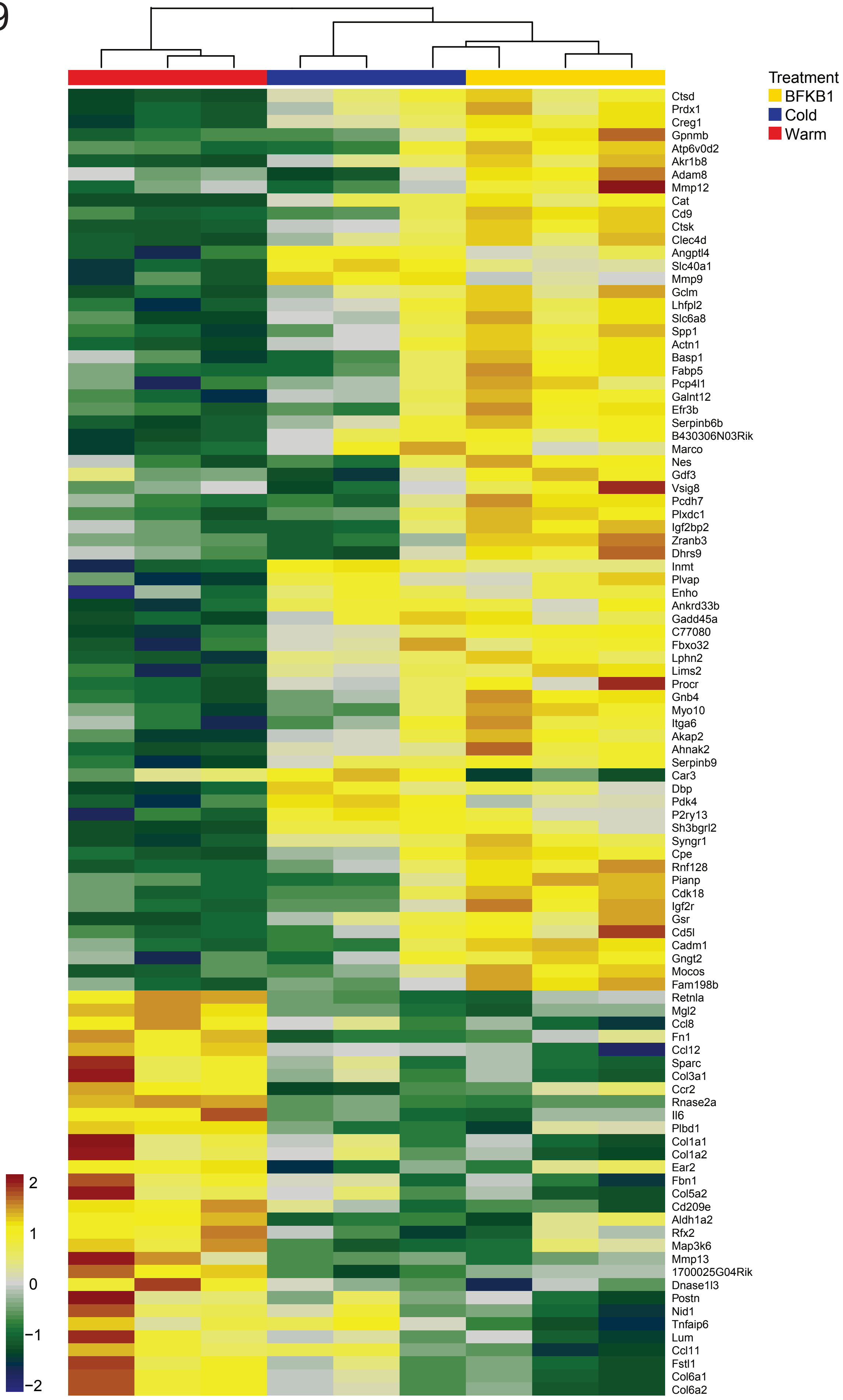
Treatment
BFKB1
Cold
Warm
2
1
0
−1
−2
Ctsd
Prdx1
Creg1
Gpnmb
Atp6v0d2
Akr1b8
Adam8
Mmp12
Cat
Cd9
Ctsk
Clec4d
Angptl4
Slc40a1
Mmp9
Gclm
Lhfpl2
Slc6a8
Spp1
Actn1
Basp1
Fabp5
Pcp4l1
Galnt12
Efr3b
Serpinb6b
B430306N03Rik
Marco
Nes
Gdf3
Vsig8
Pcdh7
Plxdc1
Igf2bp2
Zranb3
Dhrs9
Inmt
Plvap
Enho
Ankrd33b
Gadd45a
C77080
Fbxo32
Lphn2
Lims2
Procr
Gnb4
Myo10
Itga6
Akap2
Ahnak2
Serpinb9
Car3
Dbp
Pdk4
P2ry13
Sh3bgrl2
Syngr1
Cpe
Rnf128
Pianp
Cdk18
Igf2r
Gsr
Cd5l
Cadm1
Gngt2
Mocos
Fam198b
Retnla
Mgl2
Ccl8
Fn1
Ccl12
Sparc
Col3a1
Ccr2
Rnase2a
Il6
Plbd1
Col1a1
Col1a2
Ear2
Fbn1
Col5a2
Cd209e
Aldh1a2
Rfx2
Map3k6
Mmp13
1700025G04Rik
Dnase1l3
Postn
Nid1
Tnfaip6
Lum
Ccl11
Fstl1
Col6a1
Col6a2

Supplemental Figure S9. Heatmap with gene names shown in Figure 6J.

Table 1. Reagent used for the study

REAGENT	SOURCE	IDENTIFIER
**Antibodies and related reagents**		
Anti-CD45 MicroBeads	Miltenyi Biotec	130-052-301
Anti-CD31 MicroBeads	Miltenyi Biotec	130-097-418
Purified Rat Anti-Mouse CD16/CD32 (Fc Block™)	BD Biosciences	553141
Anti-CD45	BioLegend	30-F11
Anti-CD31	BioLegend	390
Anti-Podoplanin	BioLegend	8.1.1
Anti-PDGFRα	BioLegend	AP5
Anti-Lyve1	BioLegend	ALY7
Anti-Lyve1	R&D Systems	223322
Anti-CD11c	BioLegend	N418
Anti-CD206	BioLegend	C068C2
Anti-Gr1	BioLegend	RB6-8C5
Anti-FceR1	BioLegend	MAR-1
Anti-Siglec-F	BDbiosciences	E50-2440
Anti-F4/80	BioLegend	BM8
Anti-CD11b	BioLegend	M1/70
Anti-CD29	BioLegend	HMb1-1
Anti-Sca-1	BioLegend	E13-161.7
Anti-Class III β-tubulin (Tubb3)	BioLegend	AA10
Anti-CD206	Abcam	ab64693
Anti-UCP1	Abcam	ab23841
Anti-TH	EMD Millipore	AB152
Anti-TH	EMD Millipore	MAB318
Anti-Smad1	Cell Signaling Technology	9743
Anti-Phospho-Smad1/5	Cell Signaling Technology	9516
Anti-mouse IgG HRP-linked	Cell Signaling Technology	7076
Anti-rabbit IgG HRP-linked	Cell Signaling Technology	7074
Anti-Lyve-1	R&D Systems	AF2125
Anti-CD206	R&D Systems	AF2535
Anti-F4/80	BioRad	MCA497G
Anti-Col1	Abcam	ab21286
Anti-αSMA	Sigma	c6198
Anti-Arg1	Abcam	ab60167
Anti-Arg1	Abcam	ab124917
Anti-TUBB3	Abcam	ab18207
Anti-Ret	Abcam	Ab134100
Anti-HSP90	Cell Signaling Technology	#4877
Anti-FGFR1/KLB bispecific antibody	Genentech	bFKB1
Anti-Ragweed (RW, isotype control)	Genentech	
Trastuzumab (Herceptin, isotype control)	Genentech	
Anti-mouse CSF1R	BioXCell	BE0213

isotype control Rat IgG2a	BioXCell	BE0089
Picro Sirius Red	Abcam	ab150681
DAPI	Thermo Scientific	62247
Hyaluronan binding protein (HABP)	EMD Millipore	#385911
**Recombinant Proteins**		
Recombinant human/mouse BMP2	R&D Systems	355-BM
Recombinant human GDNF	R&D Systems	212-GD
Recombinant human His-GDF15, CHO	Genentech	PUR121320
Recombinant human Fc-GDF15, HEK293T	Genentech	PUR125052
**qPCR primers**		
TBP_F: ATCAACATCTCAGCAACCCA<br>TBP_R: TTGAAGCTGCGGTACAATTC	Genentech	N/A
Ppargc1a_F: CCCTGCCATTGTTAAGACC<br>Ppargc1a_R: TGCTGCTGTTCCTGTTTTC	Genentech	N/A
Scd1_F: CTGTACGGGATCATACTGGTTC<br>Scd1_R: GCCGTGCCTTGTAAGTTCTG	Genentech	N/A
Rpl13A_F: GAAGGCATCAACATTTCTGGAA<br>Rpl13A_R: CAGTGCGCCAGAAAATGC	IDT	Mm.PT.58.43547045.g
Tbp_F: TGTATCTACCGTGAATCTTGGC<br>Tbp_R: CCAGAACTGAAAATCAACGCAG	IDT	Mm.PT.39a.22214839
Ppargc1a_F: TGCAGCCAAGACTCTGTATG<br>Ppargc1a_R: TCAGAAAGGTCAAGTTCAGGAAG	IDT	Mm.PT.58.28716430
Ucp1_F: CAAATCAGCTTTGCCTCACTC<br>Ucp1_R: CACACCTCCAGTCATTAAGCC	IDT	Mm.PT.58.7088262
**Plasmid DNA**		
pFA2-ELK1	Agilent	219062
pFR-luc	Agilent	219050
pRL-SV40	Promega	E2231
CMV-Human Ret51	Genentech	J4006
CMV-Human Gfral	Genentech	DNA751304
CMV-Human Gfra1	Genentech	DNA365770
